# Improvement in work productivity among psoriatic arthritis patients treated with biologic or targeted synthetic drugs: a systematic literature review and meta-analysis

**DOI:** 10.1186/s13075-024-03282-0

**Published:** 2024-02-15

**Authors:** Laure Gossec, Brittany Humphries, Megan Rutherford, Vanessa Taieb, Damon Willems, William Tillett

**Affiliations:** 1 Sorbonne Université, INSERM, Institut Pierre Louis d’Epidémiologie et de Santé Publique, Paris, France; 2 Rheumatology Department, Pitié-Salpêtrière Hospital, AP-HP, 47-83 Bd de l’Hôpital, Paris, 75013 France; 3 Department of Health Research Methods, Evidence and Impact, McMaster University, Hamilton, Canada; 4 Cytel Inc, Ottawa, Canada; 5 Cytel Inc, London, UK; 6 UCB Pharma, Colombes, France; 7 UCB Pharma, Brussels, Belgium; 8 Department of Life Sciences, Royal National Hospital for Rheumatic Diseases, Centre for Therapeutic Innovation University of Bath, Bath, UK

**Keywords:** Psoriatic arthritis, Work productivity, WPAI, Productivity cost

## Abstract

**Background:**

Capacity to work is impacted by psoriatic arthritis (PsA). Our objective was to describe the course of work productivity and leisure activity in patients with PsA treated with biologic (b) and targeted synthetic (ts) disease-modifying antirheumatic drugs (DMARDs).

**Methods:**

A systematic literature review identified all trials and observational studies published January 1, 2010–October 22, 2021, reporting work productivity using the Work Productivity and Activity Impairment Questionnaire (WPAI) in patients with PsA treated with b/tsDMARDs. Outcomes for WPAI domains (absenteeism, presenteeism, total work productivity, and activity impairment) were collected at baseline and time point closest to 24 weeks of treatment. A random effects meta-analysis of single means was conducted to calculate an overall absolute mean change from baseline for each WPAI domain.

**Results:**

Twelve studies (ten randomized controlled and two observational) assessing patients treated with adalimumab, bimekizumab, guselkumab, ixekizumab, risankizumab, secukinumab, or upadacitinib were analysed. Among 3741 employed patients, overall mean baseline scores were 11.4%, 38.7%, 42.7%, and 48.9% for absenteeism, presenteeism, total work productivity impairment, and activity impairment, respectively. Estimated absolute mean improvements (95% confidence interval) to week 24 were 2.4 percentage points (%p) (0.6, 4.1), 17.8%p (16.2,19.3), 17.6%p (15.9,19.4), and 19.3%p (17.6, 21.0) respectively, leading to a mean relative improvement of 41% for total work productivity. The change in work outcomes in the b/tsDMARDs appeared similar.

**Conclusions:**

This systematic literature review and meta-analysis confirmed that patients with active PsA have a substantially reduced capacity to work and participate in leisure activities. Substantial improvements across various WPAI domains were noted after 24 weeks of b/tsDMARD treatment, especially in presenteeism, total work productivity, and activity impairment. These findings may be useful for reimbursement purposes and in the context of shared decision-making.

**Key summary points:**

This systematic literature review (SLR) of randomized clinical trials and observational studies of biologic (b) and targeted synthetic (ts) disease-modifying antirheumatic drugs b/tsDMARDs in patients with PsA found that at treatment introduction, patients presented with a 42.7% mean productivity loss per week as assessed by the Work Productivity and Activity Impairment (WPAI) Questionnaire.

Through a meta-analysis comparing before/after values without adjustment for placebo response, we found that after 24 weeks of treatment with b/tsDMARDs, there was a mean absolute improvement of 17.6 percentage points and a mean relative improvement of 41% in total work productivity, with similar magnitudes of improvement in time spent at work and regular activities outside of work.

These results provide clinical-, regulatory- and reimbursement decision-makers with data on the potential societal and socio-economic benefits of b/tsDMARDs in PsA.

**Supplementary Information:**

The online version contains supplementary material available at 10.1186/s13075-024-03282-0.

## Plain language summary

Psoriatic arthritis (PsA) has a major impact on patients’ lives, including their ability to work by causing absence and reducing productivity. By pooling together published studies (12 studies, corresponding to 3741 patients) and comparing what patients reported before starting treatment to during treatment, we found that over the course of treatment with biologic (b) and targeted synthetic (ts) disease-modifying antirheumatic drugs (DMARDs), patients with PsA had an average of 18% higher total work productivity, translating to a 41% reduced impact of PsA at the group level (without looking at comparisons to a placebo response). It is important for health professionals and patients to know that work outcomes affected by PsA are improved when patients take b/tsDMARDS.

**Supplementary Information:**

The online version contains supplementary material available at 10.1186/s13075-024-03282-0.

## Introduction

Psoriatic arthritis (PsA) has a substantial impact on health-related quality of life (HRQoL) [1]. As the clinical presentation of PsA varies, its impact often extends beyond joint damage to include comorbidities such as obesity, depression, anxiety, and cardiovascular disease [2, 3]. In particular, patients with PsA have reported its effect on individual activities and social participation, as well as physiological functioning as linked to the World Health Organization (WHO) International Classification of Functioning, Disability, and Health (ICF) [4, 5]. The consequences of PsA on work are important and include deleterious effects such as hours of missed work (absenteeism), diminished productivity while at work (presenteeism), and increased economic burden due to indirect costs [4, 6–10].

The availability of biologic (b) and targeted synthetic (ts) disease-modifying antirheumatic drugs (DMARDs) over the last 20 years has improved clinical outcomes in PsA [11]. However, work and work productivity following b/tsDMARD treatment initiation in PsA based on evidence from both randomized controlled trials and observational studies has not been comprehensively assessed.

The objective of this systematic literature review (SLR) and meta-analysis was to describe work and work productivity in patients with PsA prior to and following b/tsDMARD treatment, and to explore the potential economic impact of changes in productivity.

## Methods

This SLR was conducted according to the methodological guidance of the Centre for Reviews and Dissemination and reporting requirements of Preferred Reporting Items for Systematic Reviews and Meta-Analyses (PRISMA) [12, 13].

### Search strategy and selection criteria

The search aimed to capture all trials of b/tsDMARDs in PsA reporting patient-reported outcomes. The eligibility criteria were defined according to Population, Intervention, Comparator, Outcome, and Study Design (PICOS) criteria as reported in Table S1. While the SLR was designed to assess HRQoL in addition to the work impact of PsA, for this analysis we only included studies reporting work impact, captured using the Work Productivity and Activity Impairment (WPAI) questionnaire [14]. Only studies reporting outcomes related to a specific intervention as listed in the eligibility criteria were included.

The search strategy was based on key terms and synonyms related to the WPAI, work (e.g. work, employment), and productivity (e.g. presenteeism, absenteeism, impact, loss, capacity). We searched MEDLINE, Embase, EconLit, and Cochrane from January 1, 2010, through the search date of October 22, 2021. A hand search was conducted in August 2022 to update the evidence base. Data sources and the full search strategy are provided in Table S2 and Table S3, respectively.

All records were screened independently by two reviewers. Disagreements on a publication’s eligibility were resolved by discussion and/or arbitration provided by a third reviewer. Data extraction of study characteristics and outcomes of included studies was performed by a single reviewer (MR) and validated by a senior member of the research team (BH).

### Outcomes

The primary outcome of interest for this analysis was health-related work impairment as measured using the WPAI, a patient-reported outcome [14].

The WPAI has four domains to assess absenteeism (the percentage of work time missed), presenteeism (the percentage of impairment experienced while at work), overall or total work productivity impairment (work impairment due to absenteeism and presenteeism), and leisure activity impairment (impairment of activities of daily living). The four domain scores are expressed as percentages, with high percentage scores indicating a high degree of impairment and less productivity over the past 7 days [14]. Only patients employed at baseline are included in the evaluation of WPAI, with the exception of the activity impairment domain which may also include non-employed patients.

We estimated changes in WPAI by comparing WPAI domains, when starting a b/tsDMARD and after around 6 months of treatment. Of note, this corresponds to a pre-post analysis but was not adjusted for placebo response (i.e. there was no comparison to improvements in the placebo group). Indeed, not all the studies had a placebo group, and to compare to placebo, a network meta-analysis would have been needed.

To explore the indirect costs attributable to PsA and the changes following treatment (again, this corresponds to ‘raw’ changes not adjusted to placebo response), we estimated a monetary value for the total productivity loss using the human capital approach, as explained below [15, 16].

### Evidence synthesis

A random effects meta-analysis of single means was performed using RStudio Version 2022.07.1 (meta package v4.17–0) [17]. The outcome of interest was the mean change in WPAI scores from baseline to the timepoint closest to 24 weeks (i.e. 24 weeks ± 4 weeks). Meta-analysis results were reported as absolute mean change from baseline in WPAI score (weighted by study sample size) and 95% confidence interval (CI), which equates to a pooled or summary estimate of the WPAI score across the included studies around 24 weeks. For illustrative purposes, relative change was also assessed. Results for absolute change are presented as percentage point (%p), whereas relative change is percent. The reference case analysis included any studies reporting WPAI outcomes as a mean change or least-squares mean (LSM) change from baseline. A scenario analysis was performed in which the adjusted means (i.e. LSM change) were excluded from the meta-analysis and only the raw values (i.e. mean change) were considered to examine internal validity.

The results from the meta-analyses were also used to estimate the indirect costs attributable to PsA [15]. For this analysis, we assigned a monetary value to lost productivity using the human capital approach, which takes the patient’s perspective by counting any hour not worked as an hour of lost productivity [16]. Domain scores were multiplied by 40 h (assuming a standard work week) to estimate the total lost productivity (total work impairment due to absenteeism and presenteeism). We then multiplied the lost hours of productivity by €29.10 [18, 19], the reported 2021 average hourly labour costs in the European Union (27 countries, from 2020) and $40.35 [20], the 2021 employer cost for employee compensation for the United States (US), to broadly capture the international nature of the studies used in this analysis.

##  Results

**Fig. 1:**
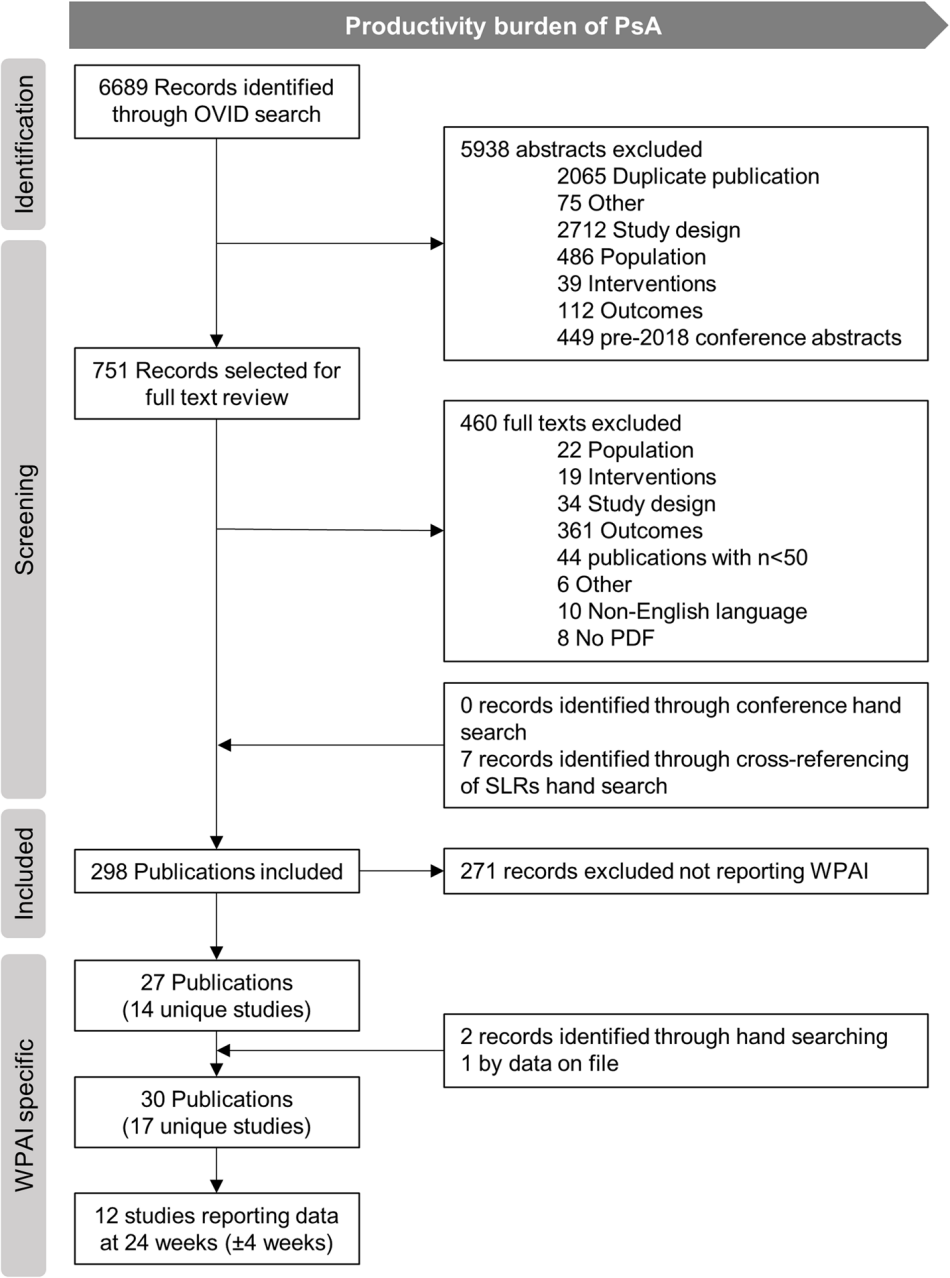
PRISMA flow of information for the systematic literature review. PRISMA, Preferred Reporting Items for Systematic Reviews and Meta-Analyses; PsA, psoriatic arthritis; WPAI, Work Productivity and Ativity Impairment Questionnaire

Of 6689 records, 751 publications were selected for full-text review, and 27 publications (from 14 unique studies) reported WPAI data. Two additional studies were identified from the hand search and one data on file from the sponsor (UCB) was also included, resulting in a total of 30 reports from 17 unique studies. Among these, 12 studies reported outcomes at a timepoint close to 24 weeks and were evaluated in the meta-analysis (Fig. 1).

Of the 12 studies, 10 drew evidence from randomized controlled trials (RCTs) with a placebo comparator and two from prospective observational studies (Table 1). Study sample sizes ranged from 100 [21] to 1281 [22] patients (mean, 532 patients). The interventions assessed included adalimumab (*n* = 4 studies), secukinumab (*n* = 3), ixekizumab (*n* = 2), risankizumab (*n* = 2), upadacitinib (*n* = 2), bimekizumab (*n* = 1), and guselkumab (*n* = 1). Most RCTs allowed patients in the intervention and placebo arms to take concomitant conventional synthetic (cs)DMARDs such as methotrexate, corticosteroids, non-steroidal anti-inflammatory drugs (NSAIDs), or other analgesics. A risk of bias assessment was performed and presented in Table S4.

**Table 1:** Study design and patient characteristics of 12 studies of bDMARDs in PsA reporting WPAI outcomes

Study name, year, and NCT	Study design, country	Intervention	Study sample size, *n*	Age (years), mean (SD)	Male, %	Disease duration (years), mean (SD)	Biologic experience, %	Employed, %	Psoriasis, %	PASI, mean (SD)	DAS28-CRP, mean (SD)	WPAI version
KEEPsAKE 1 2022 (NCT03675308) [23]	RCT, Multiple	Risankizumab 150 mg	483	51.3 (12.2)	52.2	7.1 (7.0)	0	–^a^	100	10.9 (10.1)	–	WPAI:PsA
		Placebo	481	51.2 (12.1)	48.6	7.1 (7.7)	0		100	10.0 (10.4)	–	
KEEPsAKE 2 2022 (NCT03671148) [24]	RCT, Multiple	Risankizumab 150 mg	224	53.1 (12.5)	44.6	8.2 (8.2)	46.9	–^a^	54.9^b^	7.7 (6.7)	–	WPAI:PsA
		Placebo	219	52.7 (12.6)	45.2	8.2 (8.3)	46.1		54.3^b^	8.4 (9.9)	–	
BE OPTIMAL 2022 (NCT03895203) [25] (data on file)	RCT, Multiple	Bimekizumab 160 mg	431	48.5 (12.6)	46.6	6.0 (7.3)	0	63	50.3^b^	8.2 (6.8)	3.8 (0.9)	WPAI:SHP
		Adalimumab 40 mg	140	49.0 (12.8)	50.7	6.1 (6.8)	0	64	48.6^b^	8.6 (7.6)	3.7 (0.9)	
		Placebo^e^	281	48.7 (11.7)	45.2	5.6 (6.5)	0	67	49.8^b^	7.9 (5.6)	3.8 (1.0)	
SELECT-PsA 1 2021 (NCT03104400) [22]	RCT, Multiple	Upadacitinib 15 mg QD	429	51.6 (12.2)	44.5	6.2 (7.4)	0	58^a^	–	9.8 (10.0)	–	WPAI
		Adalimumab 40 mg EOW	429	51.4 (12.0)	48.2	5.9 (7.1)	0		–	9.4 (8.5)	–	
		Placebo	423	50.4 (12.2)	51.1	6.2 (7.0)	0		–	11.2 (11.4)	–	
DISCOVER-2 2021 (NCT03158285) [26]	RCT, Multiple	Guselkumab 100 mg Q4W	245	46.0 (12.0)	58.0	5.5 (5.9)	0	62	100	10.8 (11.7)	–	WPAI:PsA
		Guselkumab 100 mg Q8W	248	45.0 (12.0)	52.0	5.1 (5.5)	0		100	9.7 (11.7)	–	
		Placebo	246	46.0 (12.0)	48.0	5.8 (5.6)	–		100	9.3 (9.8)	–	
SELECT-PsA 2 2021 (NCT03104374) [27]	RCT, Multiple	Upadacitinib 15 mg QD	211	53.0 (12.0)	46.4	9.6 (8.4)	100	–^a^	–	10.1 (9.2)	–	WPAI
		Placebo	212	54.1 (11.5)	43.4	11.0 (10.3)	100		–	11.7 (11.4)	–	
FUTURE 1 2017 (NCT01392326) [28]	RCT, Multiple	Secukinumab 150 mg Q4W	202	49.6 (11.8)	47.5	–	29.2c	51	53.5^b^	15.6 (13.9)	4.8 (1.1)	WPAI:GH
		Placebo	202	48.5 (11.2)	47.5	–		61	54.0^b^	15.1 (11.6)	4.9 (1.1)	
SPIRIT-P2 2017 (NCT02349295) [29, 30]	RCT, Multiple	Ixekizumab 80 mg Q4W	122	52.6 (13.6)	52.0	11 (9.6)	100	53	97.0^d^	6.4 (7.9)	5.1 (1.1)	WPAI:SHP
		Ixekizumab 80 mg Q2W	123	51.7 (11.9)	41.0	9.9 (7.4)	100	57	92.0^d^	6.2 (8.7)	5.1 (1.1)	
		Placebo	118	51.5 (10.4)	47.0	9.2 (7.3)	100	44.9	–	5.2 (6.3)	5.0 (1.1)	
SPIRIT-P1 2018 (NCT01695239) [29, 31]	RCT, Multiple	Adalimumab 40 mg Q2W	101	48.6 (12.4)	50.5	6.9 (7.5)	0	61	96.0^d^	5.5 (6.5)	4.9 (1.0)	WPAI:SHP
		Ixekizumab 80 mg Q4W	107	49.1 (10.1)	42.1	6.2 (6.4)	0	68	93.5^d^	6.9 (6.6)	5.0 (1.0)	
		Ixekizumab 80 mg Q2W	103	49.8 (12.6)	46.6	7.2 (8.0)	0	60	92.2^d^	6.0 (7.0)	5.0 (1.0)	
		Placebo	106	50.6 (12.3)	45.3	6.3 (6.9)	0	45.3	–	6.2 (7.5)	4.9 (1.0)	
FUTURE 2 2015 (NCT01752634) [32]	RCT, Multiple	Secukinumab 300 mg Q4W	100	46.9 (12.6)	51.0	7.4 (7.5)	33.0^c^	–^a^	41.0^b^	11.9 (8.4)^b^	4.8 (1.1)	WPAI:GH
		Secukinumab 150 mg Q4W	100	46.5 (11.7)	55.0	6.5 (8.2)	37.0^c^		58.0^b^	16.2 (14.3)^b^	4.9 (1.1)	
		Placebo	98	49.9 (12.5)	39.8	7.3 (7.8)	35.7^c^		43.9^b^	11.6 (8.3)	4.7 (1.0)	
Nakagawa 2019 (NCT02414633) [33]	Prospective observational, Japan	Adalimumab 40 mg, 80 mg	106	49.3 (10.7)	72.6	–	10.4	100	–	9.0 (8.6)	3.8 (1.3)	WPAI:PsA v2
Corrona 2020 (NCT02530268) [21]	Prospective observational, United States	Secukinumab 150 mg, 300 mg	100	51.6 (11.6)	54.3	7.0 (7.0)	83.0	–	–	–	–	WPAI

Only studies with data available at baseline and 24 weeks are included in this table

*bDMARD*, biologic disease-modifying anti-rheumatic drug; *DAS28-CRP*, Disease Activity Score 28-joint count using C reactive protein; *EOW*, every other week; *GH*, general health; *PsA*, psoriatic arthritis; *PASI*, Psoriatic Arthritis Severity Index; *Q2W*, every 2 weeks; *Q4W*, every 4 weeks; *Q8W*, every 8 weeks; *QD*, daily; *RCT*, randomized controlled trial; *SD*, standard deviation; *SHP*, Specific Health Problem; *TNFi*, tumour necrosis factor inhibitor; *WPAI*, Work Productivity and Activity Impairment

^a^Study only reports that patients employed at baseline were evaluated for all domains except activity impairment

^b^Psoriasis affecting ≥ 3% of body surface area

^c^Defined as previous number of TNFi

^d^Current psoriasis at baseline

^e^Placebo patients switched to intervention at 16 weeks

Overall, across all WPAI domains between 3683 and 5774 patients at baseline and 2425 and 3774 patients at week 24 were analysed. Among all studies, 48.8% of patients were male, the weighted mean age of patients was 50.1 years, and the weighted mean disease duration was 7.0 years (Table 1). Where reported, an average of 58.7% of patients were employed at baseline.

### Absenteeism

**Fig. 2:**
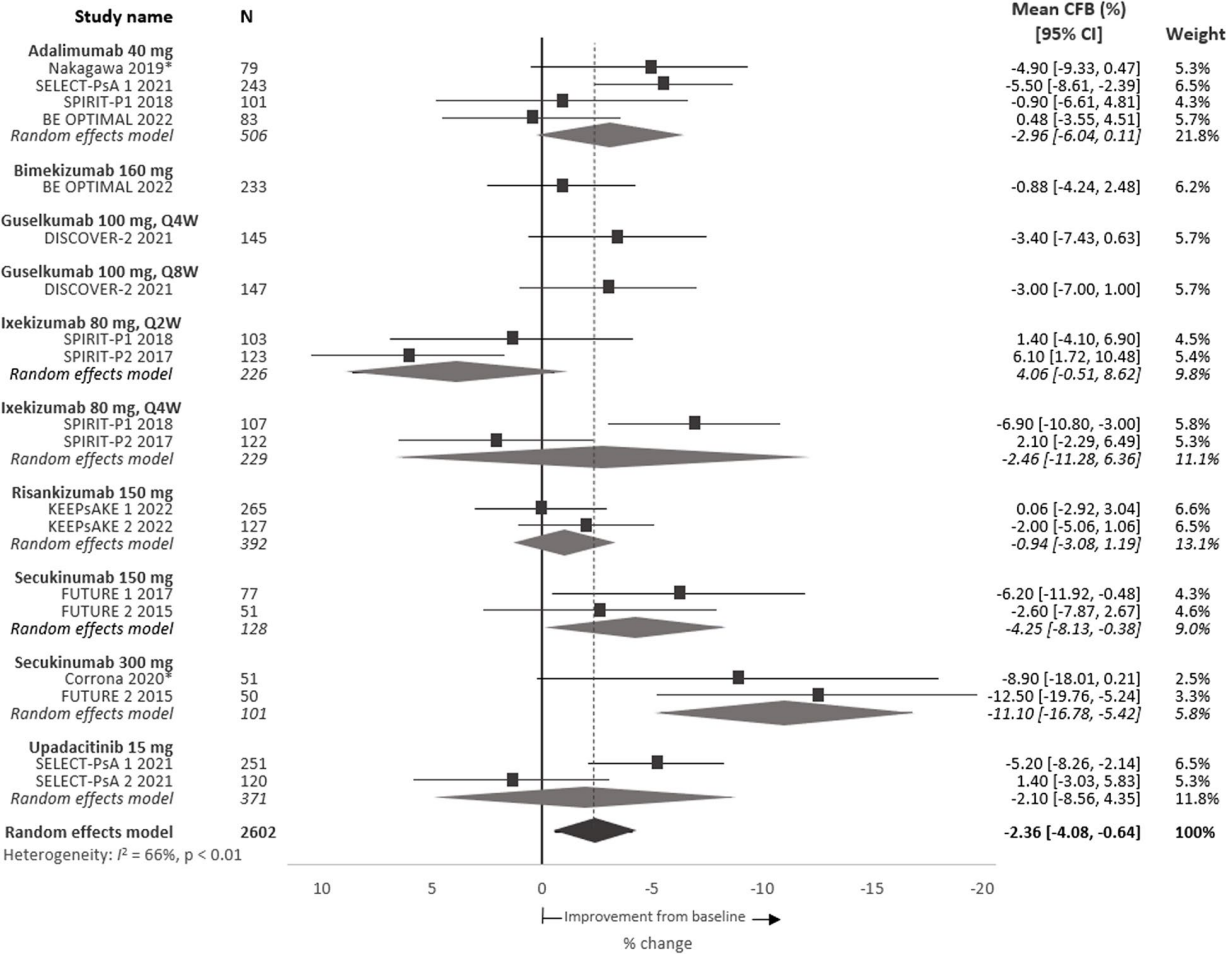
Meta-analysis of percent mean CFB in absenteeism scores per intervention at 24 weeks. Note: Point estimates represent the mean CFB scores reported for each intervention. No direct comparison is intended nor was made across interventions. *Denotes observational study. *CFB*, change from baseline;* CI*, confidence interval; *Q2W*, every 2 weeks; *Q4W*, every 4 weeks; *Q8W*, every 8 weeks

Among the 3741 patients with baseline WPAI data included in the meta-analysis, the pooled estimated mean absenteeism score (percent of time missed from work over a 7-day period due to PsA) was 11.4%, range 5.8–16.3% (95% CI 10.2, 12.6) (Table 2). This can be illustrated as 4.6 h absent from work per week, based on a 40-h work week. At week 24, the pooled absolute mean change from baseline among patients treated with a b/tsDMARD was − 2.4%p, range − 12.5 to 6.1%p (95% CI − 4.1, − 0.6) (Fig. 2), i.e. a relative improvement of 21.0%. In general, there was a greater estimated improvement in absenteeism scores among patients receiving a b/tsDMARD compared to patients taking placebo (Figure S1).

**Table 2:** Work impairment in patients with PsA, assessed through mean WPAI scores at baseline

Study name	Mean score at baseline % (SD)							
	***N***	**Absenteeism**	***N***	**Presenteeism**	***N***	**Work productivity**	***N***	**Activity impairment**
***Adalimumab 40 mg***								
Nakagawa 2019 [33]	106	8.4	106	37.5	106	40.2	104	41.7
SELECT-PsA 1 2021 [22]	243	12.8 (26.4)	230	38.3 (24.4)	243	44.8 (28.8)	429	49.3 (25.9)
SPIRIT-P1 2018 [29, 31]	101	8.7 (21.4)	101	37.3 (24.5)	101	40.6 (25.2)	101	46.9 (26.0)
BE OPTIMAL 2022 (data on file)	90	5.8 (19.4)	87	34.1 (25.5)	87	35.3 (26.3)	140	45.5 (23.5)
***Bimekizumab 160 mg***								
BE OPTIMAL 2022 (data on file)	270	7.7 (21.4)	262	34.8 (25.7)	262	37.0 (27.2)	430	43.2 (24.4)
***Ixekizumab 80 mg***								
SPIRIT-P1 2018^a^ [29, 31]	107	9.2 (21.0)	107	40.0 (26.7)	107	42.3 (28.5)	107	47.9 (26.3)
SPIRIT-P1 2018^b^ [29, 31]	103	7.7 (23.0)	103	35.8 (21.6)	103	37.4 (21.6)	103	47.1 (23.4)
SPIRIT-P2 2017^a^ [29, 30]	122	11.6 (26.6)	122	45.0 (25.7)	122	46.9 (26.7)	122	53.9 (24.9)
SPIRIT-P2 2017^b^ [29, 30]	123	8.8 (23.2)	123	36.9 (25.0)	123	38.8 (26.6)	123	49.3 (26.5)
***Risankizumab 150 mg***								
KEEPsAKE 1 2022 [23]	265	15.4 (28.6)	249	42.7 (25.5)	265	49.9 (29.9)	482	52.6 (25.1)
KEEPsAKE 2 2022 [24]	127	12.4 (24.1)	123	41.3 (26.0)	127	47.4 (28.9)	224	50.5 (26.6)
***Secukinumab 150 mg***								
FUTURE 1 2017 [28]	94	15.0 (28.4)	88	40.1 (26.7)	89	47.0 (29.8)	195	50.9 (26.4)
FUTURE 2 2015 [32]	60	7.0 (15.5)	61	37.1 (26.4)	60	39.1 (28.2)	99	48.8 (26.5)
***Secukinumab 300 mg***								
Corrona 2020 [21]	51	14.4	58	30.5	51	40.7	89	42.7
FUTURE 2 2015 [32]	63	17.0 (27.6)	59	38.1 (25.6)	59	42.7 (28.7)	99	51.0 (24.8)
***Upadacitinib 15 mg***								
SELECT-PsA 1 2021 [22]	251	11.7 (24.5)	240	43.0 (25.6)	251	48.3 (29)	429	52.0 (25.2)
SELECT-PsA 2 2021 [27]	120	15.8 (29.1)	113	41.1 (24.4)	120	48.5 (29.6)	211	52.0 (26.1)
***Placebo***								
FUTURE 1 2017 [28]	108	15.2 (26.6)	107	47.3 (28.9)	105	47.3 (28.9)	199	51.3 (26.4)
FUTURE 2 2015 [32]	59	12.4 (27.0)	57	32.5 (23.0)	55	35.4 (24.9)	98	45.1 (27.4)
SELECT-PsA 1 2021 [22]	241	16.3 (28.2)	241	43.6 (24.8)	241	50.8 (29.0)	423	49.6 (25.0)
SELECT-PsA 2 2021 [27]	100	16.3 (27.5)	95	41.9 (27.3)	100	49.4 (31.5)	212	55.1 (26.5)
SPIRIT-P1 2018 [29, 31]	106	8.9 (24.5)	106	32.4 (21.2)	106	34.6 (23.4)	106	46.1 (24.7)
SPIRIT-P2 2017 [29, 30]	118	11.9 (28.1)	118	40.4 (28.8)	118	41.5 (29.6)	118	54.0 (25.8)
KEEPsAKE 1 2022 [22]	251	12.1 (24.9)	237	39.9 (24.0)	251	46.6 (27.7)	477	52.0 (24.4)
KEEPsAKE 2 2022 [24]	136	11.8 (23.5)	131	45.1 (24.2)	136	50.1 (27.6)	219	51.6 (25.7)
BE OPTIMAL 2022 (data on file)	189	8.5 (22.1)	181	32.3 (24.7)	181	34.2 (26.3)	281	43.2 (24.5)

Some studies did not report SDs; only the mean is entered here

*SD*, standard deviation; *WPAI*, Work Productivity and Activity Impairment Questionnaire

^a^Ixekizumab 80 mg every 4 weeks

^b^Ixekizumab 80 mg every 2 weeks

### Presenteeism

The meta-analysis estimated mean presenteeism score (percent of impairment while working due to PsA) at baseline was 38.7% (*n* = 3620), range 30.5–47.3% (95% CI 35.7, 43.2) (Table 2). This equates to 15.5 h of impaired or reduced work performance per week.

**Fig. 3:**
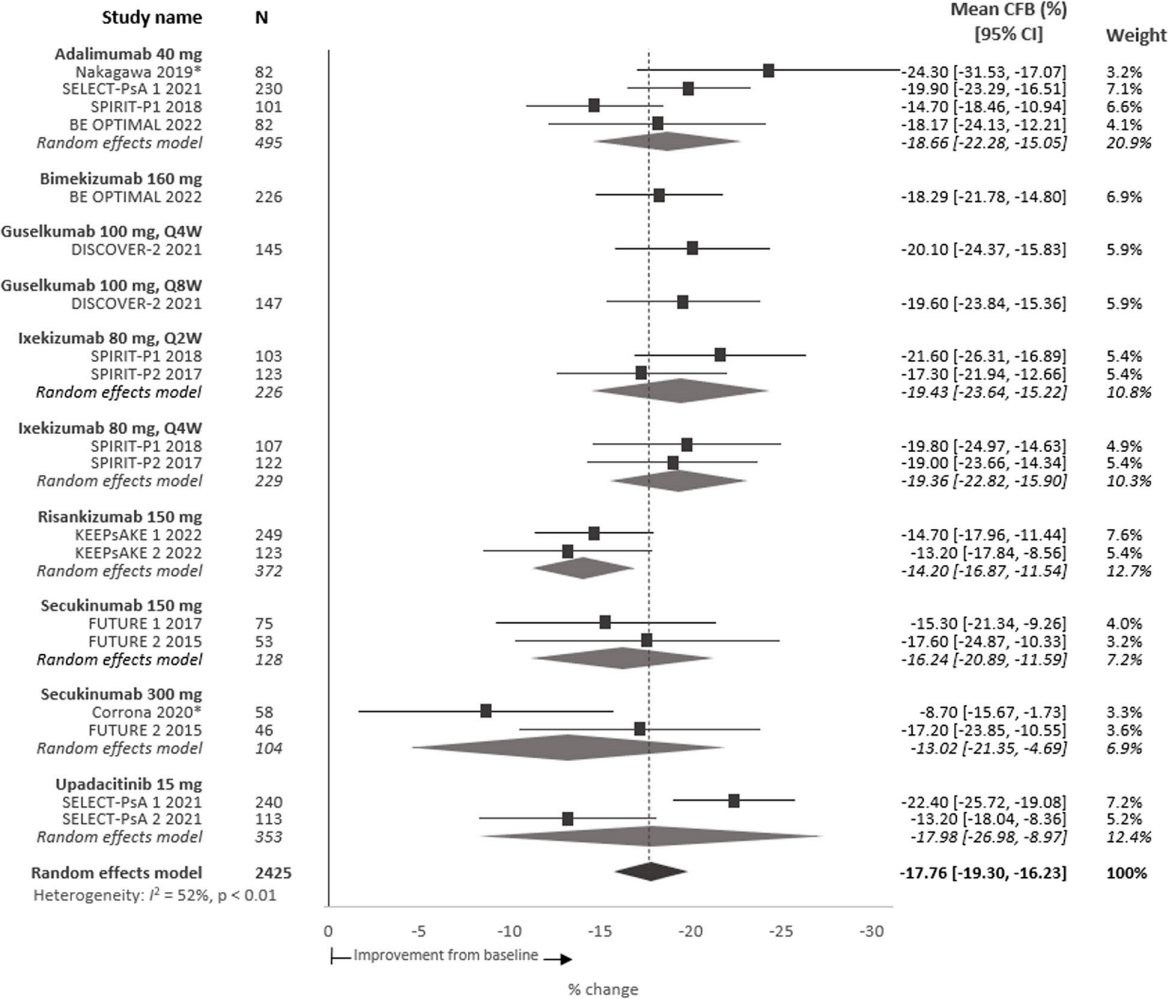
Meta-analysis of percent mean CFB in presenteeism scores per intervention at 24 weeks. Note: Point estimates represent the mean CFB scores reported for each intervention. No direct comparison is intended nor was made across interventions. *Denotes observational study.* CFB*, change from baseline; *CI*, confidence interval; *Q2W*, every 2 weeks; *Q4W*, every 4 weeks; *Q8W*, every 8 weeks

In the 12 studies (2425 patients), the pooled absolute mean change from baseline in presenteeism scores was − 17.8%p, range − 24.3 to − 8.7%p (95% CI − 19.3, − 16.2) among patients treated with a b/tsDMARD (Fig. 3) with a relative improvement of 46.0%. Improvements in presenteeism scores among patients taking placebo were smaller, with an estimated pooled mean change of − 5.5% from baseline (Figure S2).

### Work productivity impairment

**Fig. 4:**
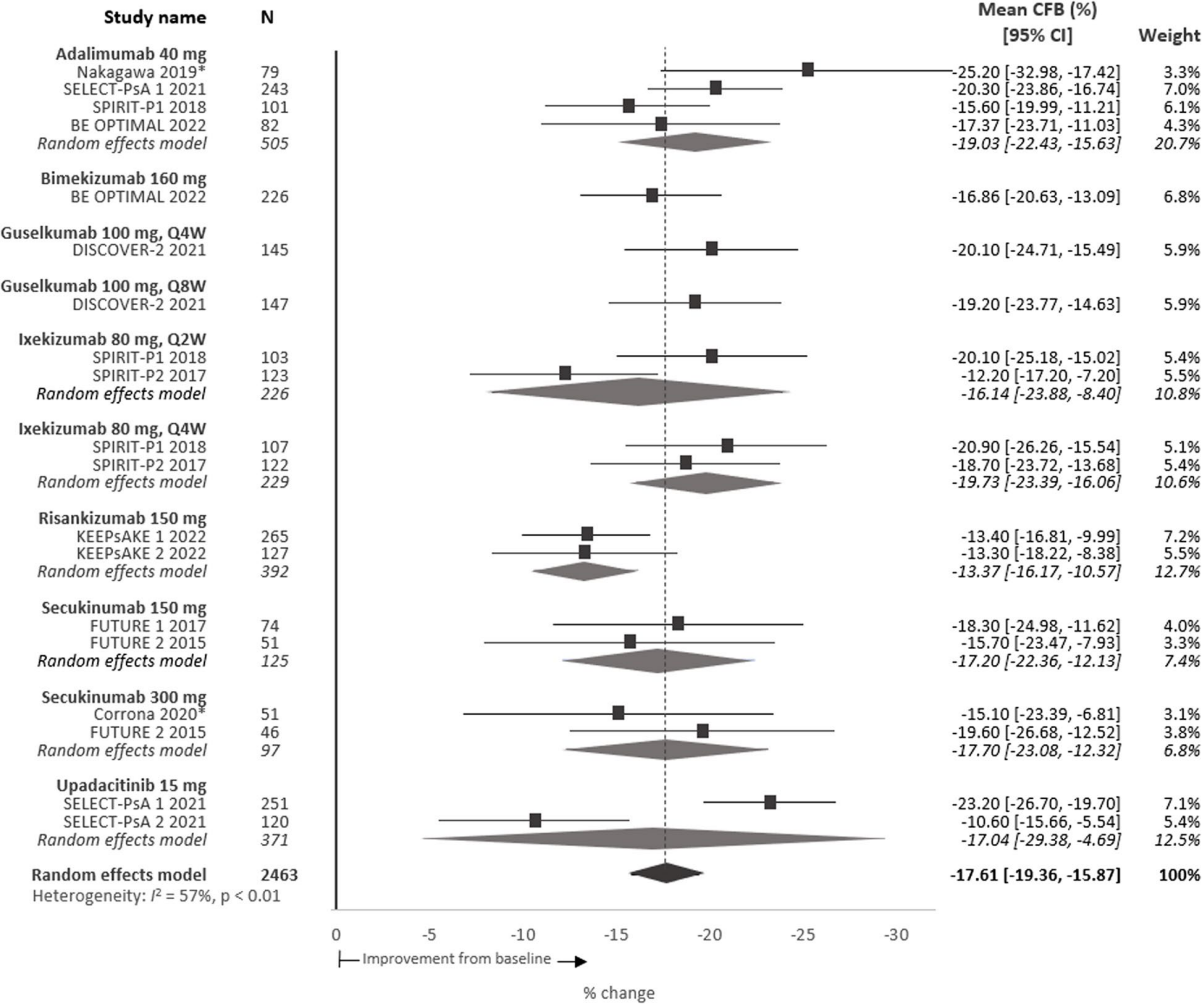
Meta-analysis of percent mean CFB total work productivity impairment scores per intervention at 24 weeks. Note: Point estimates represent the mean CFB scores reported for each intervention. No direct comparison is intended nor was made across interventions. *Denotes observational study. *CFB*, change from baseline; *CI*, confidence interval; *Q2W*, every 2 weeks; *Q4W*, every 4 weeks; *Q8W*, every 8 weeks

Baseline work productivity impairment scores were available for 3683 patients. The meta-analysis estimated mean loss of work productivity at baseline was 42.7%, range 34.2–50.8% (95% CI 40.6, 44.9) (Table 2), which can be estimated as 17.1 h of total work productivity lost per week. At 24 weeks, for patients treated with a b/tsDMARD, the pooled absolute mean change in total work productivity was − 17.6%p, range − 25.2 to − 12.2%p (95% CI − 19.4, − 15.9) (Fig. 4) with a mean relative improvement of 41.2%. Similar to other WPAI domains, improvements in total work productivity scores among patients taking placebo for up to 24 weeks were small (Figure S3).

### Activity impairment

**Fig. 5:**
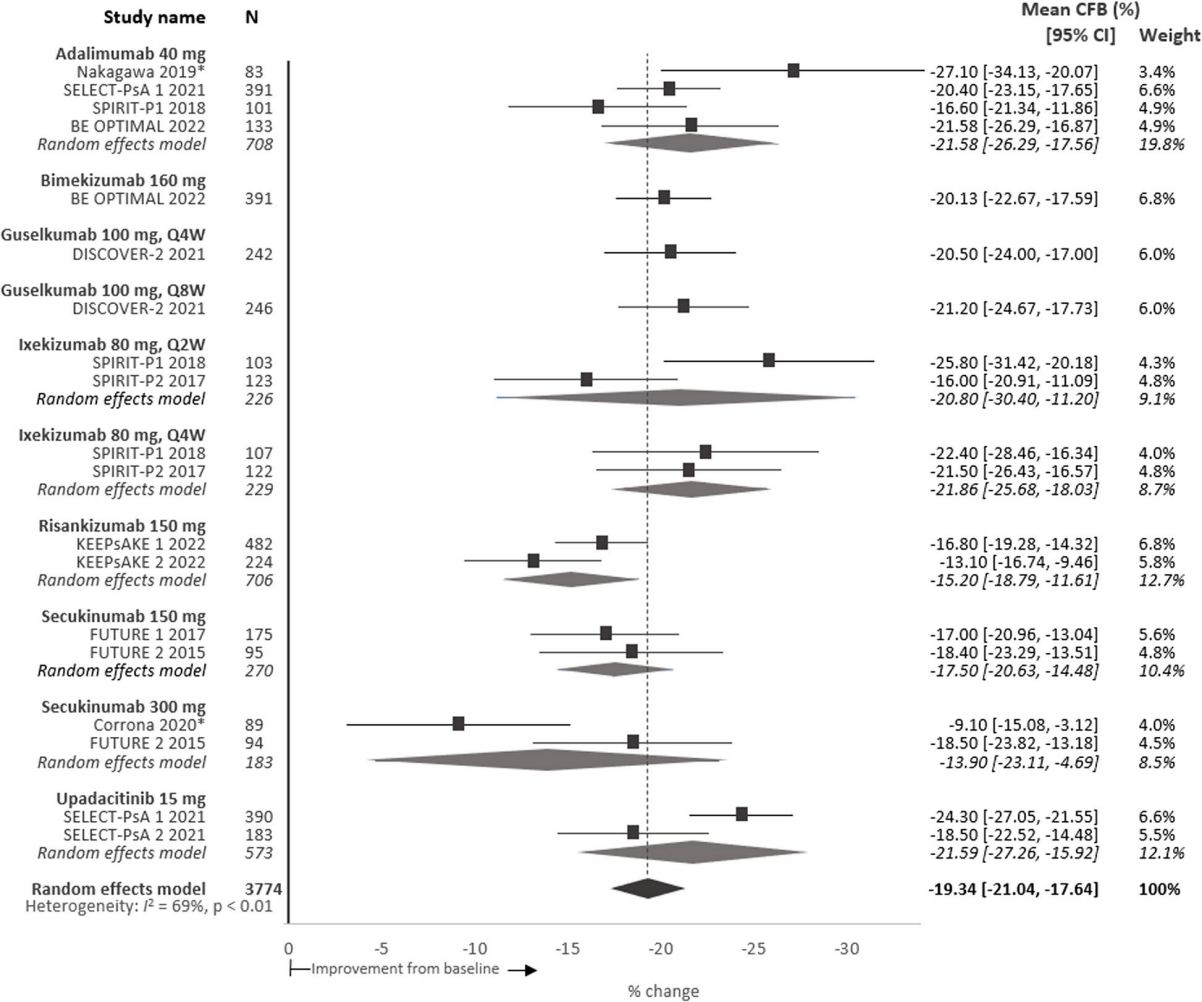
Meta-analysis of percent mean CFB in activity impairment scores per intervention at 24 weeks. Note: Point estimates represent the mean CFB scores reported for each intervention. No direct comparison is intended nor was made across interventions. *Denotes observational study. *CFB*, change from baseline; *CI*, confidence interval; *Q2W*, every 2 weeks; *Q4W*, every 4 weeks; *Q8W*, every 8 weeks

Baseline activity impairment was reported for 5774 patients, since unlike the other WPAI domains, this domain includes patients who were not employed. The estimated mean baseline impairment was 48.9%, range 41.7–55.1% (95% CI 47.5, 50.4), indicating the percent of impaired or reduced ability to participate in leisure activities over a 7-day period (Table 2). Overall, all patients treated with b/tsDMARDs reported a reduction in activity impairment from baseline with a pooled absolute mean change of − 19.3%p (95% CI − 21.4, − 17.6) (Fig. 5) and a mean relative improvement of 39.5%.

###  Indirect costs

At baseline, the overall productivity loss among patients with PsA reported in this review ranged from 13.7 to 20.3 h per week. This equates to an estimated range of indirect costs for the European Union of €20,757 to €30,833 per patient per year (US$28,782 to US$42,753). After treatment with a b/tsDMARD, there was a pooled mean improvement in total work productivity of 7.0 h per week (mean change from baseline, − 17.6%). Based on the analysis of change from baseline among patients treated with b/tsDMARDs, and without adjusting on placebo response, the estimated absolute mean decrease in PsA-related indirect costs linked to productivity was €10,688 (US$14,820) per patient, per year.

## Discussion

This review reports important information on the burden of PsA on work prior to and following b/tsDMARD treatment. In studies of b/tsDMARDs among patients with PsA, at treatment introduction, patients presented with a high burden of their disease on work, with an estimated mean work productivity loss of 17.1 h per week, or a mean reduction of 42.7% in total work productivity. In all studies, presenteeism was a greater contributor to overall work productivity loss than absenteeism. Exploratory extrapolations of indirect costs associated with work productivity impairment yielded estimates between €20,000 to €30,000 (US$28,000 to US$42,000) per person annually. Beyond work outcomes, the additional burden was noted in the form of leisure activity impairment (mean, 48.9%). Through a meta-analysis corresponding to changes with treatment (without adjustments for placebo response), we found that after 24 weeks of treatment with b/tsDMARDs, there was a mean absolute improvement of 17.6%p in total work productivity, corresponding to a mean relative improvement of 41%. After treatment with a b/tsDMARD, the pooled mean improvement in total work productivity of 7.0 h per week led to an estimated absolute mean decrease in PsA-related indirect costs of €10,688 (US$14,820) per patient, per year. These results provide clinical-, regulatory- and reimbursement decision-makers with valuable data on the societal and socio-economic benefits of b/tsDMARDs in PsA.

The findings of our review are consistent with previous studies, confirming the significant impact of PsA on work outcomes [7] and how presenteeism is a higher contributor to overall lost work productivity than absenteeism among patients with PsA [15]. This highlights that when patients with PsA attend work, their productivity is considerably impacted by PsA [34]. The reported effects of diminished productivity include reduced personal and professional development. Furthermore, as work plays an important role in one’s social life and integration into society, reduced ability to participate in work may increase isolation and have deleterious effects on the wellbeing of patients; decreased work productivity has been linked to decrements in QoL and mental health [35–37].

PsA has a high cost for society. Published estimations of the annual direct PsA-related health care costs have been reported to be as high as US$1.9 billion [8]. Indirect costs are estimated to be even greater, accounting for 52 to 72% of total disease-related costs [8]. In a systematic review and meta-analysis [38], Kawalec and colleagues estimated that the average annual indirect costs of PsA range from US$1694 to $12,318 (using the friction cost approach) or from US$1751 to $50,271 (using the human capital approach). When using the human capital approach, this range of estimates is higher than our review and can be attributed to differences in the included studies and the scope of indirect costs calculated.

Overall, improvements in productivity were significant and clinically relevant after 24 weeks of b/tsDMARDs, though variation in different domains was observed. It is important to note that the results presented here for improvements after treatment were not adjusted for changes in a comparator or placebo arm; therefore, these improvements correspond to a ‘simple’ before-after analysis and cannot in any way be considered as due to treatment (no causality is claimed). Findings for the absenteeism domain presented mixed results, with several studies not reporting an improvement in scores at 24 weeks. In contrast, all studies reported an improvement in presenteeism at 24 weeks, with a pooled estimated mean change from baseline of − 17.8% p (95% CI − 19.3, − 16.2). This is close to the minimal clinically important difference (MCID) of 20%, which was estimated by Tillett and colleagues [39]. This reported MCID applies to patient-level data; however, it provides a useful benchmark for our review.

Previous studies have demonstrated associations between productivity and response (minimal disease activity [MDA] or low disease activity according to PsA Disease Activity Score [PASDAS]) [10, 40] and found greater improvements in productivity with bDMARDs than with csDMARDs [41]. Generally, the estimated effects of the b/tsDMARDs in the current analysis were similar with respect to improving a patient’s capacity to work and participate in leisure activities. However, it is important to note that we did not directly compare the drugs as this would necessitate specific statistics such as network meta-analyses, which can provide rankings of relative effects of different treatments, but may also be influenced by heterogeneity between studies, leading to limitations in interpretability in some cases. Studies aimed to measure both the effectiveness and the impact of specific interventions on productivity and HRQoL in patients with PsA may be valuable in supporting optimized treatment selection from a holistic perspective.

This review has several strengths. The evidence was retrieved through a systematic search of the literature according to methodological guidance and reporting and included a recent update to account for the evolving treatment landscape in PsA. Analyses were based on both RCTs and observational studies, allowing for a wider scope than a previous review on this topic, which was limited to RCTs and included only five studies [42].

Despite these strengths, this review has some limitations. The analysis focused solely on the WPAI, a widely used measure of productivity that has been validated for use among patients with PsA [14, 43]. While there is no gold standard measure for assessing productivity in PsA, the WPAI was one of six instruments identified by the OMERACT Worker Productivity Group as a candidate for assessing work productivity based on available evidence regarding psychometric properties (e.g. test–retest reliability, construct validity) [44]. However, other questionnaires, such as the Work Productivity Scale (WPS) [45] or the Work Limitations Questionnaire (WLQ) [43, 46], may also be used to evaluate work impact. While this review excludes studies using these alternative assessment tools, the selection of a single measure facilitated the comparability of findings and allowed us to conduct a quantitative synthesis of findings. A network meta-analysis was not performed in which comparative treatment effects were estimated. Our approach to the analysis was taken to allow for the inclusion of observational studies that did not have a placebo arm; thus, a limitation is that no causal conclusions on the effect of b/tsDMARDs on work productivity can be derived. Future research using an NMA approach and limiting study inclusion to RCTs may provide more robust estimates.

The variation in reporting of WPAI outcomes required certain assumptions for the meta-analysis. For example, data reported as means and LSM were pooled following a scenario analysis to explore the internal validity of using raw and adjusted means. No major differences were observed between these analyses. As WPAI is often considered a secondary outcome measure, there was a lack of reporting of subgroup analyses related to patient characteristics (e.g. tumour necrosis factor inhibitors experience) and other factors that may contribute to work and/or activity impairment (e.g. pain, fatigue, participation in manual work). This limited our ability to conduct sensitivity analysis or additional analyses on subgroups of interest. This limitation reflects a shortcoming in the body of published evidence and not the methodology of the review itself.

A final limitation is the variation in the methods of estimating the costs attributable to lost productivity due to PsA. While the WPAI focuses on absenteeism and presenteeism, other components of productivity may be considered, such as early retirement due to disease or patients returning to work after successful treatment [38]. Lost productivity can also be valued using different approaches, with indirect costs typically calculated using the friction cost method or the human capital approach [16]. We estimated indirect costs using the human capital approach, which may overestimate the indirect costs incurred by employers given the limited amount of published data available. Cost conclusions are further limited by the level of heterogeneity across studies. However, these estimates are a reflection of the larger, societal burden of PsA.

Our review focused on patients with PsA treated with a b/tsDMARD. These patients could be considered as having a more severe form of disease due to failure of first-line treatment with NSAIDs and csDMARDs. Future research could consider productivity among a broader PsA population [47, 48].

The review was also structured to consider both RCT and observational evidence. Only two observational studies provided WPAI scores at 24 weeks. These studies reported some of the largest [33] and lowest [21] improvements in mean change from baseline WPAI domain scores. This suggests potential variation in productivity outcomes in real-life settings. It is important to note that one study was from the US and the other from Japan, which may have inherent differences in working and productivity. However, it is not feasible to explore this potential “country-effect” due to limited evidence and the fact that both studies had limited sample sizes; therefore, the results should be interpreted with caution.

## Conclusion

Measuring work impact should be considered an essential part of the overall assessment of the economic burden and the value assessment of therapies in PsA. This review systematically and comprehensively quantified the impact of PsA on work productivity and impairment using the WPAI among patients treated with a b/tsDMARD. The results demonstrate that patients with PsA suffered from substantial total work productivity impairment but report meaningful improvement after 24 weeks of treatment with a b/tsDMARD. This provides payers and other decision-makers with valuable data to inform decisions about the cost-effectiveness of b/tsDMARDs in PsA.

### Supplementary Information

**Additional file 1: Table S1.** PICOS eligibility criteria. **Figure S1.** Meta-analysis of percent mean CFB in absenteeism scores for placebo at 24 weeks. **Figure S2.** Meta-analysis of percent mean CFB in presenteeism scores for placebo at 24 weeks. **Figure S3.** Meta-analysis of percent mean CFB in total work productivity scores for placebo at 24 weeks. **Figure S4.** Meta-analysis of percent mean CFB activity impairment scores for placebo at 24 weeks. **Table S2.** Data sources. **Table S3.** Search strategy with results. **Table S4.** Risk of bias assessment.

## Data Availability

The data that support the findings of this study are primarily from published literature. Data from BE OPTIMAL (NCT03895203) [25] may be requested by qualified researchers 6 months after product approval in the US and/or Europe, or global development is discontinued, and 18 months after trial completion. Investigators may request access to anonymized individual patient-level data and redacted trial documents which may include analysis-ready datasets, study protocol, annotated case report form, statistical analysis plan, dataset specifications, and clinical study report. Prior to the use of the data, proposals need to be approved by an independent review panel at www.Vivli.org and a signed data-sharing agreement will need to be executed. All documents are available in English only, for a pre-specified time, typically 12 months, on a password-protected portal. This plan may change if the risk of re-identifying trial participants is determined to be too high after the trial is completed; in this case and to protect participants, individual patient-level data would not be made available.

## Abbreviations

%p: Percentage point
b: Biologic
CI: Confidence interval
cs: Conventional synthetic
DMARDs: Disease-modifying antirheumatic drugs
HRQoL: Health-related quality of life
ICF: International Classification of Functioning, Disability and Health
LSM: Least-squares mean
MCID: Minimal clinically important difference
MDA: Minimal disease activity
NSAID: Non-steroidal anti-inflammatory drugs
PASDAS: Psoriatic Arthritis Disease Activity Score
PICOS: Population, Intervention, Comparator, Outcome and Study Design
PRISMA: Preferred Reporting Items for Systematic Reviews and Meta-Analyses
PsA: Psoriatic arthritis
RCT: Randomized controlled trials
SLR: Systematic literature review
ts: Targeted synthetic
WHO: World Health Organization
WLQ: Work Limitations Questionnaire
WPAI: Work Productivity and Activity Impairment Questionnaire
WPS: Work Productivity Scale
