# Supplementary material for: Improvement in work productivity among psoriatic arthritis patients treated with biologic or targeted synthetic drugs: a systematic literature review and meta-analysis
Source: Arthritis Res Ther. 2024 Feb 15;26:50. doi: 10.1186/s13075-024-03282-0 (PMC10868000; doi:10.1186/s13075-024-03282-0)
Supplement: Supplementary file 1 — Additional file 1: Table S1. PICOS eligibility criteria. Figure S1. Meta-analysis of percent mean CFB in absenteeism scores for placebo at 24 weeks. Figure S2. Meta-analysis of percent mean CFB in presenteeism scores for placebo at 24 weeks. Figure S3. Meta-analysis of percent mean CFB in total work productivity scores for placebo at 24 weeks. Figure S4. Meta-analysis of percent mean CFB activity impairment scores for placebo at 24 weeks. Table S2. Data sources. Table S3. Search strategy with results. Table S4. Risk of bias assessment. [file 13075_2024_3282_MOESM1_ESM.docx]

Supplementary Material

Table S1. PICOS eligibility criteria

| **Dimension** | **Criteria** |
| --- | --- |
| Patient population | Psoriatic arthritis (adults only) |
| Interventions | Abatacept |
|  | Adalimumab |
|  | Apremilast |
|  | Bimekizumab |
|  | Brodalumab |
|  | Certolizumab pegol |
|  | Etanercept |
|  | Golimumab |
|  | Guselkumab |
|  | Infliximab |
|  | Ixekizumab |
|  | Risankizumab |
|  | Secukinumab |
|  | Tildrakizumab |
|  | Tofacitinib |
|  | Upadacitinib |
|  | Ustekinumab |
| Comparators | Standard of care (NSAIDs, DMARDs) |
|  | Placebo |
| Outcomes  (specific to WPAI) | Work Productivity and Activity Impairment Questionnaire (WPAI) |
|  | WPAI: General Health (WPAI:GH) |
|  | WPAI: GH v2 |
|  | WPAI: Psoriatic Arthritis (WPAI:PsA) |
|  | WPAI: PsA v2 |
|  | WPAI: Specific Health Problem (WPAI:SHP) |
|  | WPAI: SHP v2 |
| Study design | Studies with following designs (but not limited to): |
|  | Real-world studies |
|  | Clinical trials |
| Publication type | Original manuscripts |
|  | Conference abstracts/posters |
| Other limits | 2010 – October 21, 2021, for original manuscripts  2018 – October 21, 2021, for conference abstracts/posters |

*DMARDs, Disease-modifying anti-rheumatic drugs; GH, General Health; NSAIDs, Non-steroidal anti-inflammatory drugs; PICOS, Population, Intervention, Comparator, Outcome and Study Design; PsA, Psoriatic Arthritis; SHP, Specific Health Problem; WPAI, Work Productivity and Activity Impairment Questionnaire.*

Figure S1. Meta-analysis of percent mean CFB in absenteeism scores for placebo at 24 weeks


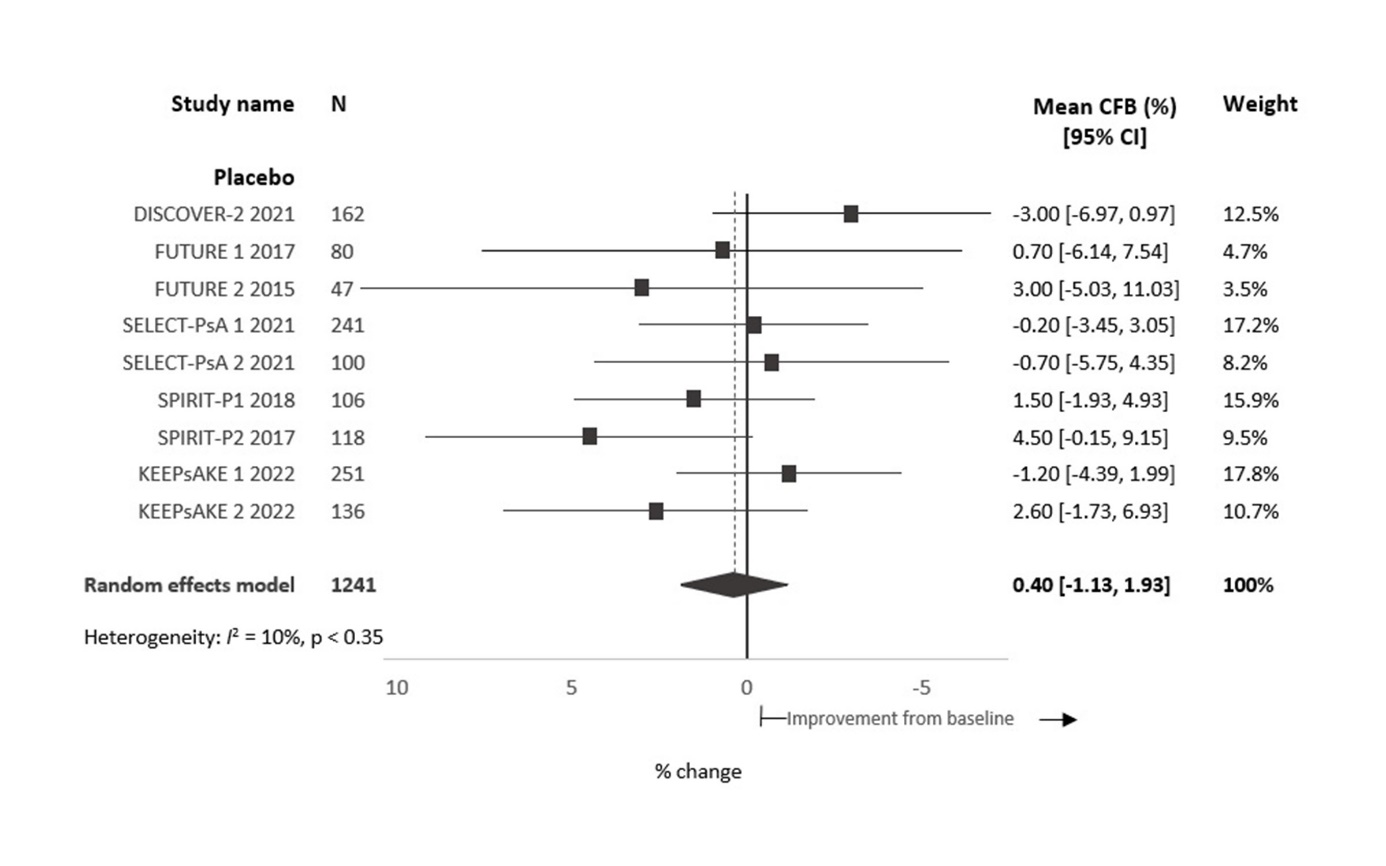
CFB, Change from baseline; CI, Confidence interval.

Figure S2. Meta-analysis of percent mean CFB in presenteeism scores for placebo at 24 weeks


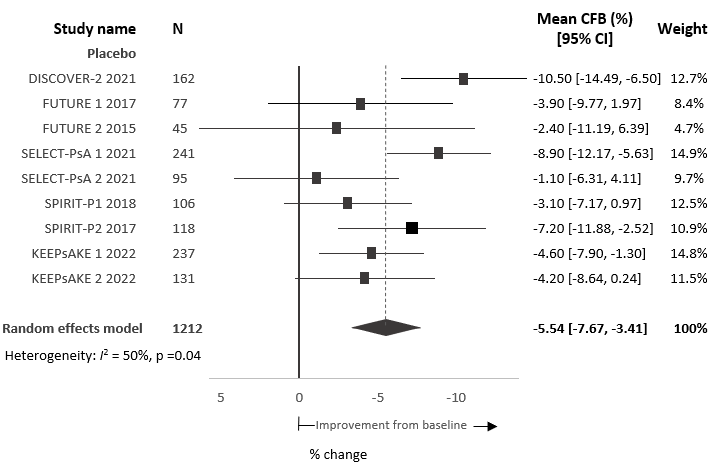


CFB, Change from baseline; CI, Confidence interval.

Figure S3. Meta-analysis of percent mean CFB in total work productivity scores for placebo at 24 weeks


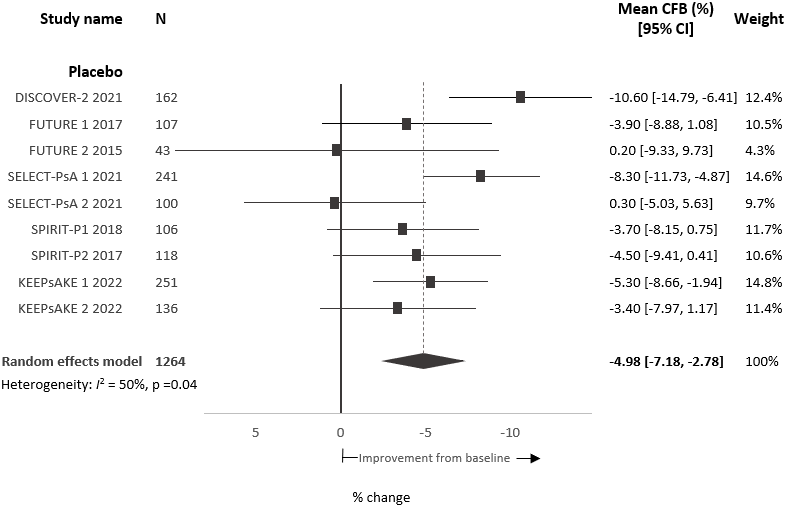


*CFB, Change from baseline; CI, Confidence interval.*

Figure S4. Meta-analysis of percent mean CFB activity impairment scores for placebo at 24 weeks


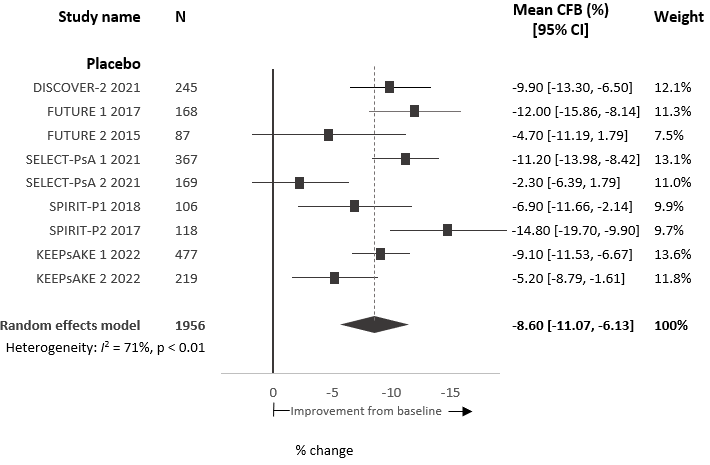


CFB, Change from baseline; CI, Confidence interval.

Table S2. Data sources

| **Data sources** | |
| --- | --- |
| Electronic databases (via Ovid.com) | - Medical Literature Analysis and Retrieval System Online (MEDLINE®) - MEDLINE® Epub Ahead of Print, In-Process & Other Non-Indexed Citations - Excerpta Medica Database (Embase®) - Cochrane   - Cochrane Central Register of Controlled Trials   - Cochrane Database of Systematic Reviews   - Cochrane Clinical Answers   - Cochrane Methodology Register |
| Conferences | - All conferences identified via the electronic database search (published in the last five years) - Hand-search of the proceedings from 2018 onwards of the following conferences (if not yet indexed in Embase at the time of the search):   - American College of Rheumatology (ACR)   - European Alliance of Associations for Rheumatology (EULAR)   - British Society for Rheumatology (BSR)   - Academy of Managed Care Pharmacy (AMCP) - The Professional Society for Health Economics and Outcomes Research (ISPOR) |
| Other sources | - Bibliography list of relevant SLRs/meta-analyses identified by the database searches |

ACR, American College of Rheumatology; AMCP, Academy of Managed Care Pharmacy; BSR, British Society for Rheumatology; Embase, Excerpta Medica Database; EULAR, European Alliance of Associations for Rheumatology; ISPOR, The Professional Society for Health Economics and Outcomes Research; MEDLINE, Medical Literature Analysis and Retrieval System Online; SLR, Systematic literature review.

Table S3. Search strategy with results

| **Search** | | **Query** | | | **Hits** | |
| --- | --- | --- | --- | --- | --- | --- |
| **MEDLINE (1946 to October 21, 2021)** | | | | | | |
| 1 | | Arthritis, Psoriatic/ | | | 6975 | |
| 2 | | psoriatic arthritis/ | | | 6975 | |
| 3 | | (psoria$ adj2 (arthrit$ or arthropath$)).ti,ab. | | | 11380 | |
| 4 | | or/1-3 | | | 12520 | |
| 5 | | Adalimumab/ or Golimumab/ or Infliximab/ or Etanercept/ or Bimekizumab/ or Secukinumab/ or Certolizumab/ or Ustekinumab/ or Tofacitinib/ or Ixekizumab/ or Tildrakizumab/ or Guselkumab/ or Risankizumab/ or Apremilast/ or Brodalumab/ or Upadacitinib/ or Abatacept/ | | | 22250 | |
| 6 | | (Humira* or Enbrel* or Remicade* or Cimzia* or Simponi* or adalimumab* or Cosentyx*).mp. | | | 10272 | |
| 7 | | (certolizumab or CDP870 or cimzia or 428863-50-7).mp. | | | 1442 | |
| 8 | | (etanercept or enbrel or altebrel or 185243-69-0).mp. | | | 9206 | |
| 9 | | (Cosentyx* or secukinumab* or AIN?457 or AIN 457).mp. | | | 1489 | |
| 10 | | (golimumab or CNTO 148 or simponi or 476181-74-5).mp. | | | 1421 | |
| 11 | | (Remicade* or Inflectra* or Flixabi* or Remsima* or Renflexis* or Ixifi* or infliximab* or TA?650 or TA 650).mp. | | | 16190 | |
| 12 | | (Enbrel* or Benepali* or Erelzi* or Lifmior* or etanercept* or TNR?001 or "TNR 001" or methotrexate).mp. | | | 64506 | |
| 13 | | (Stelara* or CNTO 1275 or CNTO-1275 or CNTO1275 or Ustekinumab).mp. | | | 2501 | |
| 14 | | (Xeljanz* or CP 690550 or CP-690550 or CP690550 or Tofacitinib).mp. | | | 2006 | |
| 15 | | (Taltz* or Ixekizumab).mp. | | | 747 | |
| 16 | | (Ilumya* or Ilumetri* or Tildrakizumab-asmn or Tildrakizumab).mp. | | | 184 | |
| 17 | | (Tremfya* or Guselkumab).mp. | | | 363 | |
| 18 | | (Skyrizi* or BI 655066 or BI-655066 or BI655066 or "ABBV 066" or ABBV-066 or ABBV066 or Risankizumab).mp. | | | 222 | |
| 19 | | (Orencia* or Abatacept).mp. | | | 4042 | |
| 20 | | (Otezla* or Aplex* or CC-10004 or CC10004 or CC 10004 or Apremilast).mp. | | | 871 | |
| 21 | | (Siliq* or Kyntheum* or KHK4827 or KHK-4827 or KHK 4827 or AMG827 or AMG-827 or AMG 827 or Brodalumab).mp. | | | 1961 | |
| 22 | | (Rinvoq* or ABT494 or ABT-494 or ABT 494 or Upadacitinib).mp. | | | 236 | |
| 23 | | or/5-22 | | | 89120 | |
| 24 | | "quality of life"/ | | | 223478 | |
| 25 | | (QOL* or HQL* or HQOL* or H QOL* or HRQL* or HRQOL* or HR QOL*).ti,ab. | | | 65225 | |
| 26 | | (quality adj4 life).ti,ab. | | | 324970 | |
| 27 | | (quality adj2 well?being).ti,ab. | | | 359 | |
| 28 | | Quality-Adjusted Life Years/ | | | 13892 | |
| 29 | | "quality adjusted life year".de. | | | 0 | |
| 30 | | "disability adjusted life".ti,ab. | | | 4033 | |
| 31 | | (qal* or qwb* or qald* or qale* or qtime* or daly*).ti,ab. | | | 16314 | |
| 32 | | health* year* equivalent*.ti,ab. | | | 40 | |
| 33 | | hye*.ti,ab. | | | 1124 | |
| 34 | | Patient Reported Outcome Measures/ | | | 9743 | |
| 35 | | patient-reported outcome/ | | | 9743 | |
| 36 | | (patient adj2 reported adj2 outcome adj2 measure$).ti,ab. | | | 7531 | |
| 37 | | (preference* adj4 (patient* or public or valu* or measur*)).ti,ab. | | | 26364 | |
| 38 | | PRO.ti,ab. | | | 215900 | |
| 39 | | (utilit* or disutilit*).ti,ab. | | | 232652 | |
| 40 | | utility measure*.ti,ab. | | | 563 | |
| 41 | | standard gamble*.ti,ab. | | | 881 | |
| 42 | | (time trade off* or time tradeoff* or timetradeoff or timetrade off*).ti,ab. | | | 1528 | |
| 43 | | (willingness adj4 pay).ti,ab. | | | 6817 | |
| 44 | | (SG or TTO or WTP).ti,ab. | | | 14706 | |
| 45 | | ((valu* or measur*) adj4 (health or outcome or outcomes or effect or effects or change* or state*)).ti,ab. | | | 554148 | |
| 46 | | (utilit* adj4 (valu* or measur* or health or life or estimat* or elicit* or disease)).ti,ab. | | | 15853 | |
| 47 | | (euroqol* or euro qol or euroqual or euro qual or eq?5d or eq 5d).ti,ab. | | | 13324 | |
| 48 | | (eq?vas or eq vas or visual analogue scale*).ti,ab. | | | 31040 | |
| 49 | | ("Health Utilities Index" or HUI*).ti,ab. | | | 5429 | |
| 50 | | (multiattribute* adj1 (health or theor* or analys* or utilit*)).ti,ab. | | | 173 | |
| 51 | | ((multi adj1 attribute*) and (attribute* adj1 theor*)).ti,ab. | | | 1 | |
| 52 | | ((multi adj1 attribute*) and (attribute* adj1 analys*)).ti,ab. | | | 12 | |
| 53 | | ((multi adj1 attribute*) and (attribute* adj1 utilit*)).ti,ab. | | | 199 | |
| 54 | | (sf?36* or sf 36 or short form 36 or shortform 36 or shortform?36).ti,ab. | | | 27780 | |
| 55 | | (sf?20* or sf 20 or short form 20 or shortform 20 or shortform?20).ti,ab. | | | 474 | |
| 56 | | (sf?12* or sf 12 or short form 12 or shortform 12 or shortform?12).ti,ab. | | | 6723 | |
| 57 | | (sf?8* or sf 8 or short form 8 or shortform 8 or shortform?8).ti,ab. | | | 901 | |
| 58 | | (sf?6* or sf 6 or short form 6 or shortform 6 or shortform?6).ti,ab. | | | 4323 | |
| 59 | | productivity/ or absenteeism/ | | | 23113 | |
| 60 | | (Productivit$ or WPAI or work limitation$).tw. | | | 68222 | |
| 61 | | (work$ or school$ or employ$).tw. | | | 2498787 | |
| 62 | | ((work or working) adj1 (absen$ or loss$ or disabilit$ or abilit$ or impairment$ or limitation$ or incapacit$ or capacit$)).tw. | | | 14984 | |
| 63 | | ((symptom or symptoms) adj5 (score* or scale* or instrument* or measur*)).ti,ab. | | | 80453 | |
| 64 | | sickness impact profile/ | | | 7268 | |
| 65 | | sickness impact profile.ti,ab. | | | 1072 | |
| 66 | | ("Psoriasis Index of Quality of Life" or PSORIQoL or "Psoriasis Life Stress Inventory" or PLSI or "Psoriasis Disability Index" or PDI or "Psoriasis Area and Severity Index" or PASI or "Simplified PASI" or "Simplified Psoriasis Area and Severity Index" or SAPASI or "Psoriasis Symptom Assessment of PSA" or "Patient's Global Psoriasis Assessment" or PGPA).tw. | | | 11521 | |
| 67 | | ("Physician* Global Assessment" or "Patient* Global Assessment" or PGA).tw. | | | 7680 | |
| 68 | | ("Questionnaire on Experience with Skin Complaints" or QES or "Dermatology Life Quality Index" or DLQI).tw. | | | 2567 | |
| 69 | | ("Salford Psoriasis Index" or SPI or "Koo-Menter Psoriasis Instrument" or KMPI).tw. | | | 3977 | |
| 70 | | ("Psoriatic Arthritis Quality of Life" or PsAQoL).tw. | | | 38 | |
| 71 | | ("Health Assessment Questionnaire" or HAQ* or "modified HAQ" or "modified Health Assessment Questionnaire" or MHAQ or "Health assessment questionnaire disability index" or HAQ?DI or HAQ DI or "Rheumatoid Arthritis Quality of Life Questionnaire" or RAQoL).tw. | | | 5779 | |
| 72 | | (Investigator* adj2 Global).mp. | | | 1066 | |
| 73 | | fatigue.mp. | | | 119935 | |
| 74 | | pain.mp. | | | 781222 | |
| 75 | | ("activit$ of daily living" or "Daily Living Activit$" or self-care$ or selfcare$ or "self care$" or self-manage$ or "self manage$" or selfmanage$ or (function$ adj6 (physical or role or emotional or social or cognitive or living or daily or activit$ or patient$ or person$ or mov$ or brain or mental))).tw. | | | 727779 | |
| 76 | | ("Dermatology Quality of Life Index" or DLQI).mp. | | | 1802 | |
| 77 | | (PHQ-9 or PHQ?9 or "Patient Health Questionnaire").mp. [mp=title, abstract, original title, name of substance word, subject heading word, floating sub-heading word, keyword heading word, organism supplementary concept word, protocol supplementary concept word, rare disease supplementary concept word, unique identifier, synonyms] | | | 8797 | |
| 78 | | ("Psoriatic Arthritis Impact of Disease" or PsAID*).mp. | | | 45 | |
| 79 | | or/24-78 | | | 5037082 | |
| 80 | | 4 and 23 and 79 | | | 1210 | |
| 81 | | (letter or editorial or comment or news or newspaper article).pt. | | | 2214262 | |
| 82 | | (addresses or bibliography or biography or case report or comment or congresses or consensus development conference or duplicate publication or editorial or guideline or in vitro or interview or lectures or letter or monograph or news or "newspaper article" or practice guideline or "review literature" or "review of reported cases" or review, academic or review, multicase or review, tutorial or twin study).pt. | | | 2478598 | |
| 83 | | animals/ not (humans/ and animals/) | | | 4865958 | |
| 84 | | or/81-83 | | | 7258554 | |
| 85 | | 80 not 84 | | | 1185 | |
| 86 | | limit 85 to human | | | 994 | |
| 87 | | limit 86 to yr="2010 -Current" | | | 727 | |
| **Embase (1946 to October 21, 2021)** | | | | | | |
| 1 | | Arthritis, Psoriatic/ | | 13452 | | |
| 2 | | psoriatic arthritis/ | | 26090 | | |
| 3 | | (psoria$ adj2 (arthrit$ or arthropath$)).ti,ab. | | 22847 | | |
| 4 | | or/1-3 | | 29516 | | |
| 5 | | Adalimumab/ or Golimumab/ or Infliximab/ or Etanercept/ or Bimekizumab/ or Secukinumab/ or Certolizumab/ or Ustekinumab/ or Tofacitinib/ or Ixekizumab/ or Tildrakizumab/ or Guselkumab/ or Risankizumab/ or Apremilast/ or Brodalumab/ or Upadacitinib/ or Abatacept/ | | 98275 | | |
| 6 | | (Humira* or Enbrel* or Remicade* or Cimzia* or Simponi* or adalimumab* or Cosentyx*).mp. | | 43416 | | |
| 7 | | (certolizumab or CDP870 or cimzia or 428863-50-7).mp. | | 8335 | | |
| 8 | | (etanercept or enbrel or altebrel or 185243-69-0).mp. | | 34941 | | |
| 9 | | (Cosentyx* or secukinumab* or AIN?457 or AIN 457).mp. | | 5204 | | |
| 10 | | (golimumab or CNTO 148 or simponi or 476181-74-5).mp. | | 8365 | | |
| 11 | | (Remicade* or Inflectra* or Flixabi* or Remsima* or Renflexis* or Ixifi* or infliximab* or TA?650 or TA 650).mp. | | 56478 | | |
| 12 | | (Enbrel* or Benepali* or Erelzi* or Lifmior* or etanercept* or TNR?001 or "TNR 001" or methotrexate).mp. | | 214199 | | |
| 13 | | (Stelara* or CNTO 1275 or CNTO-1275 or CNTO1275 or Ustekinumab).mp. | | 9228 | | |
| 14 | | (Xeljanz* or CP 690550 or CP-690550 or CP690550 or Tofacitinib).mp. | | 6505 | | |
| 15 | | (Taltz* or Ixekizumab).mp. | | 2511 | | |
| 16 | | (Ilumya* or Ilumetri* or Tildrakizumab-asmn or Tildrakizumab).mp. | | 694 | | |
| 17 | | (Tremfya* or Guselkumab).mp. | | 1271 | | |
| 18 | | (Skyrizi* or BI 655066 or BI-655066 or BI655066 or "ABBV 066" or ABBV-066 or ABBV066 or Risankizumab).mp. | | 718 | | |
| 19 | | (Orencia* or Abatacept).mp. | | 10752 | | |
| 20 | | (Otezla* or Aplex* or CC-10004 or CC10004 or CC 10004 or Apremilast).mp. | | 2817 | | |
| 21 | | (Siliq* or Kyntheum* or KHK4827 or KHK-4827 or KHK 4827 or AMG827 or AMG-827 or AMG 827 or Brodalumab).mp. | | 3048 | | |
| 22 | | (Rinvoq* or ABT494 or ABT-494 or ABT 494 or Upadacitinib).mp. | | 954 | | |
| 23 | | or/5-22 | | 265748 | | |
| 24 | | "quality of life"/ | | 527020 | | |
| 25 | | (QOL* or HQL* or HQOL* or H QOL* or HRQL* or HRQOL* or HR QOL*).ti,ab. | | 117647 | | |
| 26 | | (quality adj4 life).ti,ab. | | 514374 | | |
| 27 | | (quality adj2 well?being).ti,ab. | | 599 | | |
| 28 | | Quality-Adjusted Life Years/ | | 30016 | | |
| 29 | | "quality adjusted life year".de. | | 30016 | | |
| 30 | | "disability adjusted life".ti,ab. | | 4842 | | |
| 31 | | (qal* or qwb* or qald* or qale* or qtime* or daly*).ti,ab. | | 28286 | | |
| 32 | | health* year* equivalent*.ti,ab. | | 41 | | |
| 33 | | hye*.ti,ab. | | 1642 | | |
| 34 | | Patient Reported Outcome Measures/ | | 31614 | | |
| 35 | | patient-reported outcome/ | | 34252 | | |
| 36 | | (patient adj2 reported adj2 outcome adj2 measure$).ti,ab. | | 11164 | | |
| 37 | | (preference* adj4 (patient* or public or valu* or measur*)).ti,ab. | | 38923 | | |
| 38 | | PRO.ti,ab. | | 327013 | | |
| 39 | | (utilit* or disutilit*).ti,ab. | | 323517 | | |
| 40 | | utility measure*.ti,ab. | | 874 | | |
| 41 | | standard gamble*.ti,ab. | | 1145 | | |
| 42 | | (time trade off* or time tradeoff* or timetradeoff or timetrade off*).ti,ab. | | 2155 | | |
| 43 | | (willingness adj4 pay).ti,ab. | | 10455 | | |
| 44 | | (SG or TTO or WTP).ti,ab. | | 22283 | | |
| 45 | | ((valu* or measur*) adj4 (health or outcome or outcomes or effect or effects or change* or state*)).ti,ab. | | 712703 | | |
| 46 | | (utilit* adj4 (valu* or measur* or health or life or estimat* or elicit* or disease)).ti,ab. | | 24419 | | |
| 47 | | (euroqol* or euro qol or euroqual or euro qual or eq?5d or eq 5d).ti,ab. | | 24475 | | |
| 48 | | (eq?vas or eq vas or visual analogue scale*).ti,ab. | | 47034 | | |
| 49 | | ("Health Utilities Index" or HUI*).ti,ab. | | 6726 | | |
| 50 | | (multiattribute* adj1 (health or theor* or analys* or utilit*)).ti,ab. | | 194 | | |
| 51 | | ((multi adj1 attribute*) and (attribute* adj1 theor*)).ti,ab. | | 2 | | |
| 52 | | ((multi adj1 attribute*) and (attribute* adj1 analys*)).ti,ab. | | 18 | | |
| 53 | | ((multi adj1 attribute*) and (attribute* adj1 utilit*)).ti,ab. | | 289 | | |
| 54 | | (sf?36* or sf 36 or short form 36 or shortform 36 or shortform?36).ti,ab. | | 45220 | | |
| 55 | | (sf?20* or sf 20 or short form 20 or shortform 20 or shortform?20).ti,ab. | | 538 | | |
| 56 | | (sf?12* or sf 12 or short form 12 or shortform 12 or shortform?12).ti,ab. | | 10793 | | |
| 57 | | (sf?8* or sf 8 or short form 8 or shortform 8 or shortform?8).ti,ab. | | 1530 | | |
| 58 | | (sf?6* or sf 6 or short form 6 or shortform 6 or shortform?6).ti,ab. | | 7305 | | |
| 59 | | productivity/ or absenteeism/ | | 58300 | | |
| 60 | | (Productivit$ or WPAI or work limitation$).tw. | | 81114 | | |
| 61 | | (work$ or school$ or employ$).tw. | | 3123361 | | |
| 62 | | ((work or working) adj1 (absen$ or loss$ or disabilit$ or abilit$ or impairment$ or limitation$ or incapacit$ or capacit$)).tw. | | 18534 | | |
| 63 | | ((symptom or symptoms) adj5 (score* or scale* or instrument* or measur*)).ti,ab. | | 124926 | | |
| 64 | | sickness impact profile/ | | 2360 | | |
| 65 | | sickness impact profile.ti,ab. | | 1224 | | |
| 66 | | ("Psoriasis Index of Quality of Life" or PSORIQoL or "Psoriasis Life Stress Inventory" or PLSI or "Psoriasis Disability Index" or PDI or "Psoriasis Area and Severity Index" or PASI or "Simplified PASI" or "Simplified Psoriasis Area and Severity Index" or SAPASI or "Psoriasis Symptom Assessment of PSA" or "Patient's Global Psoriasis Assessment" or PGPA).tw. | | 18457 | | |
| 67 | | ("Physician* Global Assessment" or "Patient* Global Assessment" or PGA).tw. | | 13450 | | |
| 68 | | ("Questionnaire on Experience with Skin Complaints" or QES or "Dermatology Life Quality Index" or DLQI).tw. | | 5254 | | |
| 69 | | ("Salford Psoriasis Index" or SPI or "Koo-Menter Psoriasis Instrument" or KMPI).tw. | | 4988 | | |
| 70 | | ("Psoriatic Arthritis Quality of Life" or PsAQoL).tw. | | 145 | | |
| 71 | | ("Health Assessment Questionnaire" or HAQ* or "modified HAQ" or "modified Health Assessment Questionnaire" or MHAQ or "Health assessment questionnaire disability index" or HAQ?DI or HAQ DI or "Rheumatoid Arthritis Quality of Life Questionnaire" or RAQoL).tw. | | 15177 | | |
| 72 | | (Investigator* adj2 Global).mp. | | 2065 | | |
| 73 | | fatigue.mp. | | 294236 | | |
| 74 | | pain.mp. | | 1386332 | | |
| 75 | | ("activit$ of daily living" or "Daily Living Activit$" or self-care$ or selfcare$ or "self care$" or self-manage$ or "self manage$" or selfmanage$ or (function$ adj6 (physical or role or emotional or social or cognitive or living or daily or activit$ or patient$ or person$ or mov$ or brain or mental))).tw. | | 1027097 | | |
| 76 | | ("Dermatology Quality of Life Index" or DLQI).mp. | | 4073 | | |
| 77 | | (PHQ-9 or PHQ?9 or "Patient Health Questionnaire").mp. [mp=title, abstract, heading word, drug trade name, original title, device manufacturer, drug manufacturer, device trade name, keyword heading word, floating subheading word, candidate term word] | | 17840 | | |
| 78 | | ("Psoriatic Arthritis Impact of Disease" or PsAID*).mp. | | 196 | | |
| 79 | | or/24-78 | | 6989849 | | |
| 80 | | 4 and 23 and 79 | | 5805 | | |
| 81 | | (letter or editorial or comment or news or newspaper article).pt. | | 1900726 | | |
| 82 | | (addresses or bibliography or biography or case report or comment or congresses or consensus development conference or duplicate publication or editorial or guideline or in vitro or interview or lectures or letter or monograph or news or "newspaper article" or practice guideline or "review literature" or "review of reported cases" or review, academic or review, multicase or review, tutorial or twin study).pt. | | 1900726 | | |
| 83 | | animals/ not (humans/ and animals/) | | 990902 | | |
| 84 | | or/81-83 | | 2875821 | | |
| 85 | | 80 not 84 | | 5678 | | |
| 86 | | limit 85 to human | | 5441 | | |
| 87 | | limit 86 to yr="2010 -Current" | | 4695 | | |
| **EBM reviews (1946 to October 21, 2021)*** | | | | | | |
| 1 | Arthritis, Psoriatic/ | | 522 | | | |
| 2 | psoriatic arthritis/ | | 522 | | | |
| 3 | (psoria$ adj2 (arthrit$ or apacityy$)).ti,ab. | | 2630 | | | |
| 4 | or/1-3 | | 2687 | | | |
| 5 | Adalimumab/ or Golimumab/ or Infliximab/ or Etanercept/ or Bimekizumab/ or Secukinumab/ or Certolizumab/ or Ustekinumab/ or Tofacitinib/ or Ixekizumab/ or Tildrakizumab/ or Guselkumab/ or Risankizumab/ or Apremilast/ or Brodalumab/ or Upadacitinib/ or Abatacept/ | | 2853 | | | |
| 6 | (Humira* or Enbrel* or Remicade* or Cimzia* or Simponi* or adalimumab* or Cosentyx*).mp. | | 4646 | | | |
| 7 | (certolizumab or CDP870 or cimzia or 428863-50-7).mp. | | 853 | | | |
| 8 | (etanercept or apaci or altebrel or 185243-69-0).mp. | | 2825 | | | |
| 9 | (Cosentyx* or secukinumab* or AIN?457 or AIN 457).mp. | | 1078 | | | |
| 10 | (golimumab or CNTO 148 or apacit or 476181-74-5).mp. | | 899 | | | |
| 11 | (Remicade* or Inflectra* or Flixabi* or Remsima* or Renflexis* or Ixifi* or infliximab* or TA?650 or TA 650).mp. | | 3258 | | | |
| 12 | (Enbrel* or Benepali* or Erelzi* or Lifmior* or etanercept* or TNR?001 or “TNR 001” or methotrexate).mp. | | 15421 | | | |
| 13 | (Stelara* or CNTO 1275 or CNTO-1275 or CNTO1275 or Ustekinumab).mp. | | 1071 | | | |
| 14 | (Xeljanz* or CP 690550 or CP-690550 or CP690550 or Tofacitinib).mp. | | 962 | | | |
| 15 | (Taltz* or Ixekizumab).mp. | | 586 | | | |
| 16 | (Ilumya* or Ilumetri* or Tildrakizumab-asmn or Tildrakizumab).mp. | | 195 | | | |
| 17 | (Tremfya* or Guselkumab).mp. | | 347 | | | |
| 18 | (Skyrizi* or BI 655066 or BI-655066 or BI655066 or “ABBV 066” or ABBV-066 or ABBV066 or Risankizumab).mp. | | 165 | | | |
| 19 | (Orencia* or Abatacept).mp. | | 1043 | | | |
| 20 | (Otezla* or Aplex* or CC-10004 or CC10004 or CC 10004 or Apremilast).mp. | | 526 | | | |
| 21 | (Siliq* or Kyntheum* or KHK4827 or KHK-4827 or KHK 4827 or AMG827 or AMG-827 or AMG 827 or Brodalumab).mp. | | 225 | | | |
| 22 | (Rinvoq* or ABT494 or ABT-494 or ABT 494 or Upadacitinib).mp. | | 426 | | | |
| 23 | or/5-22 | | 23775 | | | |
| 24 | “quality of life”/ | | 27498 | | | |
| 25 | (QOL* or HQL* or HQOL* or H QOL* or HRQL* or HRQOL* or HR QOL*).ti,ab. | | 28329 | | | |
| 26 | (quality adj4 life).ti,ab. | | 120226 | | | |
| 27 | (quality adj2 well?being).ti,ab. | | 239 | | | |
| 28 | Quality-Adjusted Life Years/ | | 4635 | | | |
| 29 | “quality adjusted life year”.de. | | 0 | | | |
| 30 | “disability adjusted life”.ti,ab. | | 275 | | | |
| 31 | (qal* or qwb* or qald* or qale* or qtime* or daly*).ti,ab. | | 4723 | | | |
| 32 | health* year* equivalent*.ti,ab. | | 1 | | | |
| 33 | hye*.ti,ab. | | 49 | | | |
| 34 | Patient Reported Outcome Measures/ | | 809 | | | |
| 35 | patient-reported outcome/ | | 0 | | | |
| 36 | (patient adj2 reported adj2 outcome adj2 measure$).ti,ab. | | 1829 | | | |
| 37 | (preference* adj4 (patient* or public or valu* or measur*)).ti,ab. | | 7270 | | | |
| 38 | PRO.ti,ab. | | 13984 | | | |
| 39 | (apacit* or apacityy*).ti,ab. | | 19079 | | | |
| 40 | utility measure*.ti,ab. | | 250 | | | |
| 41 | standard gamble*.ti,ab. | | 112 | | | |
| 42 | (time trade off* or time tradeoff* or timetradeoff or timetrade off*).ti,ab. | | 241 | | | |
| 43 | (willingness adj4 pay).ti,ab. | | 1669 | | | |
| 44 | (SG or TTO or WTP).ti,ab. | | 2031 | | | |
| 45 | ((valu* or measur*) adj4 (health or outcome or outcomes or effect or effects or change* or state*)).ti,ab. | | 178037 | | | |
| 46 | (apacit* adj4 (valu* or measur* or health or life or apacity* or elicit* or disease)).ti,ab. | | 3820 | | | |
| 47 | (euroqol* or euro qol or euroqual or euro qual or eq?5d or eq 5d).ti,ab. | | 10731 | | | |
| 48 | (eq?vas or eq vas or visual analogue scale*).ti,ab. | | 26342 | | | |
| 49 | (“Health Utilities Index” or HUI*).ti,ab. | | 565 | | | |
| 50 | (multiattribute* adj1 (health or theor* or analys* or apacit*)).ti,ab. | | 15 | | | |
| 51 | ((multi adj1 attribute*) and (attribute* adj1 theor*)).ti,ab. | | 0 | | | |
| 52 | ((multi adj1 attribute*) and (attribute* adj1 analys*)).ti,ab. | | 1 | | | |
| 53 | ((multi adj1 attribute*) and (attribute* adj1 utilit*)).ti,ab. | | 24 | | | |
| 54 | (sf?36* or sf 36 or short form 36 or shortform 36 or shortform?36).ti,ab. | | 14061 | | | |
| 55 | (sf?20* or sf 20 or short form 20 or shortform 20 or shortform?20).ti,ab. | | 88 | | | |
| 56 | (sf?12* or sf 12 or short form 12 or shortform 12 or shortform?12).ti,ab. | | 2950 | | | |
| 57 | (sf?8* or sf 8 or short form 8 or shortform 8 or shortform?8).ti,ab. | | 314 | | | |
| 58 | (sf?6* or sf 6 or short form 6 or shortform 6 or shortform?6).ti,ab. | | 1619 | | | |
| 59 | productivity/ or absenteeism/ | | 912 | | | |
| 60 | (Productivit$ or WPAI or work limitation$).tw. | | 5728 | | | |
| 61 | (work$ or school$ or employ$).tw. | | 155784 | | | |
| 62 | ((work or working) adj1 (absen$ or loss$ or apacityy$ or apacit$ or impairment$ or limitation$ or apacityy$ or apacity$)).tw. | | 4087 | | | |
| 63 | ((symptom or symptoms) adj5 (score* or scale* or instrument* or measur*)).ti,ab. | | 43828 | | | |
| 64 | sickness impact profile/ | | 571 | | | |
| 65 | sickness impact profile.ti,ab. | | 298 | | | |
| 66 | (“Psoriasis Index of Quality of Life” or PSORIQoL or “Psoriasis Life Stress Inventory” or PLSI or “Psoriasis Disability Index” or PDI or “Psoriasis Area and Severity Index” or PASI or “Simplified PASI” or “Simplified Psoriasis Area and Severity Index” or SAPASI or “Psoriasis Symptom Assessment of PSA” or “Patient’s Global Psoriasis Assessment” or PGPA).tw. | | 3728 | | | |
| 67 | (“Physician* Global Assessment” or “Patient* Global Assessment” or PGA).tw. | | 4433 | | | |
| 68 | (“Questionnaire on Experience with Skin Complaints” or QES or “Dermatology Life Quality Index” or DLQI).tw. | | 1937 | | | |
| 69 | (“Salford Psoriasis Index” or SPI or “Koo-Menter Psoriasis Instrument” or KMPI).tw. | | 371 | | | |
| 70 | (“Psoriatic Arthritis Quality of Life” or PsAQoL).tw. | | 40 | | | |
| 71 | (“Health Assessment Questionnaire” or HAQ* or “modified HAQ” or “modified Health Assessment Questionnaire” or MHAQ or “Health assessment questionnaire disability index” or HAQ?DI or HAQ DI or “Rheumatoid Arthritis Quality of Life Questionnaire” or RAQoL).tw. | | 4218 | | | |
| 72 | (Investigator* adj2 Global).mp. | | 1978 | | | |
| 73 | fatigue.mp. | | 40859 | | | |
| 74 | pain.mp. | | 213568 | | | |
| 75 | (“activit$ of daily living” or “Daily Living Activit$” or self-care$ or selfcare$ or “self care$” or self-manage$ or “self manage$” or selfmanage$ or (function$ adj6 (physical or role or emotional or social or cognitive or living or daily or activit$ or patient$ or person$ or mov$ or brain or mental))).tw. | | 141846 | | | |
| 76 | (“Dermatology Quality of Life Index” or DLQI).mp. | | 1535 | | | |
| 77 | (PHQ-9 or PHQ?9 or “Patient Health Questionnaire”).mp. [mp=ti, ab, tx, kw, ct, ot, sh, hw] | | 3710 | | | |
| 78 | (“Psoriatic Arthritis Impact of Disease” or PsAID*).mp. | | 29 | | | |
| 79 | or/24-78 | | 686937 | | | |
| 80 | 4 and 23 and 79 | | 1358 | | | |
| 81 | (letter or editorial or comment or news or newspaper article).pt. | | 9152 | | | |
| 82 | (addresses or bibliography or biography or case report or comment or congresses or consensus development conference or duplicate publication or editorial or guideline or in vitro or interview or lectures or letter or monograph or news or “newspaper article” or practice guideline or “review literature” or “review of reported cases” or review, academic or review, multicase or review, tutorial or twin study).pt. | | 9355 | | | |
| 83 | animals/ not (humans/ and animals/) | | 21 | | | |
| 84 | or/81-83 | | 9376 | | | |
| 85 | 80 not 84 | | 1358 | | | |
| 86 | limit 85 to human [Limit not valid in CDSR,ACP Journal Club,DARE,CCA,CCTR,CLCMR; records were retained] | | 1357 | | | |
| 87 | limit 86 to yr=”2010 -Current” [Limit not valid in DARE; records were retained] | | 1265 | | | |
| **EconLit (1946 to October 21, 2021)**** | | | | | | |
| 1 | [Arthritis, Psoriatic/] | | | | | 0 |
| 2 | [psoriatic arthritis/] | | | | | 0 |
| 3 | (psoria$ adj2 (arthrit$ or arthropath$)).ti,ab. | | | | | 5 |
| 4 | or/1-3 | | | | | 5 |
| 5 | [Adalimumab/ or Golimumab/ or Infliximab/ or Etanercept/ or Bimekizumab/ or Secukinumab/ or Certolizumab/ or Ustekinumab/ or Tofacitinib/ or Ixekizumab/ or Tildrakizumab/ or Guselkumab/ or Risankizumab/ or Apremilast/ or Brodalumab/ or Upadacitinib/ or Abatacept/] | | | | | 0 |
| 6 | (Humira* or Enbrel* or Remicade* or Cimzia* or Simponi* or adalimumab* or Cosentyx*).mp. | | | | | 8 |
| 7 | (certolizumab or CDP870 or cimzia or 428863-50-7).mp. | | | | | 1 |
| 8 | (etanercept or enbrel or altebrel or 185243-69-0).mp. | | | | | 10 |
| 9 | (Cosentyx* or secukinumab* or AIN?457 or AIN 457).mp. | | | | | 1 |
| 10 | (golimumab or CNTO 148 or simponi or 476181-74-5).mp. | | | | | 3 |
| 11 | (Remicade* or Inflectra* or Flixabi* or Remsima* or Renflexis* or Ixifi* or infliximab* or TA?650 or TA 650).mp. | | | | | 16 |
| 12 | (Enbrel* or Benepali* or Erelzi* or Lifmior* or etanercept* or TNR?001 or "TNR 001" or methotrexate).mp. | | | | | 14 |
| 13 | (Stelara* or CNTO 1275 or CNTO-1275 or CNTO1275 or Ustekinumab).mp. | | | | | 1 |
| 14 | (Xeljanz* or CP 690550 or CP-690550 or CP690550 or Tofacitinib).mp. | | | | | 0 |
| 15 | (Taltz* or Ixekizumab).mp. | | | | | 0 |
| 16 | (Ilumya* or Ilumetri* or Tildrakizumab-asmn or Tildrakizumab).mp. | | | | | 0 |
| 17 | (Tremfya* or Guselkumab).mp. | | | | | 0 |
| 18 | (Skyrizi* or BI 655066 or BI-655066 or BI655066 or "ABBV 066" or ABBV-066 or ABBV066 or Risankizumab).mp. | | | | | 0 |
| 19 | (Orencia* or Abatacept).mp. | | | | | 0 |
| 20 | (Otezla* or Aplex* or CC-10004 or CC10004 or CC 10004 or Apremilast).mp. | | | | | 1 |
| 21 | (Siliq* or Kyntheum* or KHK4827 or KHK-4827 or KHK 4827 or AMG827 or AMG-827 or AMG 827 or Brodalumab).mp. | | | | | 0 |
| 22 | (Rinvoq* or ABT494 or ABT-494 or ABT 494 or Upadacitinib).mp. | | | | | 0 |
| 23 | or/5-22 | | | | | 25 |
| 24 | ["quality of life"/] | | | | | 0 |
| 25 | (QOL* or HQL* or HQOL* or H QOL* or HRQL* or HRQOL* or HR QOL*).ti,ab. | | | | | 278 |
| 26 | (quality adj4 life).ti,ab. | | | | | 4166 |
| 27 | (quality adj2 well?being).ti,ab. | | | | | 13 |
| 28 | [Quality-Adjusted Life Years/] | | | | | 0 |
| 29 | ["quality adjusted life year".de.] | | | | | 0 |
| 30 | "disability adjusted life".ti,ab. | | | | | 97 |
| 31 | (qal* or qwb* or qald* or qale* or qtime* or daly*).ti,ab. | | | | | 741 |
| 32 | health* year* equivalent*.ti,ab. | | | | | 16 |
| 33 | hye*.ti,ab. | | | | | 47 |
| 34 | [Patient Reported Outcome Measures/] | | | | | 0 |
| 35 | [patient-reported outcome/] | | | | | 0 |
| 36 | (patient adj2 reported adj2 outcome adj2 measure$).ti,ab. | | | | | 12 |
| 37 | (preference* adj4 (patient* or public or valu* or measur*)).ti,ab. | | | | | 2875 |
| 38 | PRO.ti,ab. | | | | | 6668 |
| 39 | (utilit* or disutilit*).ti,ab. | | | | | 32542 |
| 40 | utility measure*.ti,ab. | | | | | 89 |
| 41 | standard gamble*.ti,ab. | | | | | 87 |
| 42 | (time trade off* or time tradeoff* or timetradeoff or timetrade off*).ti,ab. | | | | | 144 |
| 43 | (willingness adj4 pay).ti,ab. | | | | | 6352 |
| 44 | (SG or TTO or WTP).ti,ab. | | | | | 2254 |
| 45 | ((valu* or measur*) adj4 (health or outcome or outcomes or effect or effects or change* or state*)).ti,ab. | | | | | 21751 |
| 46 | (utilit* adj4 (valu* or measur* or health or life or estimat* or elicit* or disease)).ti,ab. | | | | | 2438 |
| 47 | (euroqol* or euro qol or euroqual or euro qual or eq?5d or eq 5d).ti,ab. | | | | | 209 |
| 48 | (eq?vas or eq vas or visual analogue scale*).ti,ab. | | | | | 58 |
| 49 | ("Health Utilities Index" or HUI*).ti,ab. | | | | | 260 |
| 50 | (multiattribute* adj1 (health or theor* or analys* or utilit*)).ti,ab. | | | | | 60 |
| 51 | ((multi adj1 attribute*) and (attribute* adj1 theor*)).ti,ab. | | | | | 1 |
| 52 | ((multi adj1 attribute*) and (attribute* adj1 analys*)).ti,ab. | | | | | 7 |
| 53 | ((multi adj1 attribute*) and (attribute* adj1 utilit*)).ti,ab. | | | | | 74 |
| 54 | (sf?36* or sf 36 or short form 36 or shortform 36 or shortform?36).ti,ab. | | | | | 43 |
| 55 | (sf?20* or sf 20 or short form 20 or shortform 20 or shortform?20).ti,ab. | | | | | 1 |
| 56 | (sf?12* or sf 12 or short form 12 or shortform 12 or shortform?12).ti,ab. | | | | | 25 |
| 57 | (sf?8* or sf 8 or short form 8 or shortform 8 or shortform?8).ti,ab. | | | | | 2 |
| 58 | (sf?6* or sf 6 or short form 6 or shortform 6 or shortform?6).ti,ab. | | | | | 17 |
| 59 | [productivity/ or absenteeism/] | | | | | 0 |
| 60 | (Productivit$ or WPAI or work limitation$).tw. | | | | | 51301 |
| 61 | (work$ or school$ or employ$).tw. | | | | | 273088 |
| 62 | ((work or working) adj1 (absen$ or loss$ or disabilit$ or abilit$ or impairment$ or limitation$ or incapacit$ or capacit$)).tw. | | | | | 383 |
| 63 | ((symptom or symptoms) adj5 (score* or scale* or instrument* or measur*)).ti,ab. | | | | | 53 |
| 64 | [sickness impact profile/] | | | | | 0 |
| 65 | sickness impact profile.ti,ab. | | | | | 1 |
| 66 | ("Psoriasis Index of Quality of Life" or PSORIQoL or "Psoriasis Life Stress Inventory" or PLSI or "Psoriasis Disability Index" or PDI or "Psoriasis Area and Severity Index" or PASI or "Simplified PASI" or "Simplified Psoriasis Area and Severity Index" or SAPASI or "Psoriasis Symptom Assessment of PSA" or "Patient's Global Psoriasis Assessment" or PGPA).tw. | | | | | 36 |
| 67 | ("Physician* Global Assessment" or "Patient* Global Assessment" or PGA).tw. | | | | | 60 |
| 68 | ("Questionnaire on Experience with Skin Complaints" or QES or "Dermatology Life Quality Index" or DLQI).tw. | | | | | 13 |
| 69 | ("Salford Psoriasis Index" or SPI or "Koo-Menter Psoriasis Instrument" or KMPI).tw. | | | | | 83 |
| 70 | ("Psoriatic Arthritis Quality of Life" or PsAQoL).tw. | | | | | 0 |
| 71 | ("Health Assessment Questionnaire" or HAQ* or "modified HAQ" or "modified Health Assessment Questionnaire" or MHAQ or "Health assessment questionnaire disability index" or HAQ?DI or HAQ DI or "Rheumatoid Arthritis Quality of Life Questionnaire" or RAQoL).tw. | | | | | 68 |
| 72 | (Investigator* adj2 Global).mp. | | | | | 1 |
| 73 | fatigue.mp. | | | | | 337 |
| 74 | pain.mp. | | | | | 748 |
| 75 | ("activit$ of daily living" or "Daily Living Activit$" or self-care$ or selfcare$ or "self care$" or self-manage$ or "self manage$" or selfmanage$ or (function$ adj6 (physical or role or emotional or social or cognitive or living or daily or activit$ or patient$ or person$ or mov$ or brain or mental))).tw. | | | | | 6116 |
| 76 | ("Dermatology Quality of Life Index" or DLQI).mp. | | | | | 1 |
| 77 | (PHQ-9 or PHQ?9 or "Patient Health Questionnaire").mp. [mp=heading words, abstract, title, country as subject] | | | | | 4 |
| 78 | ("Psoriatic Arthritis Impact of Disease" or PsAID*).mp. | | | | | 0 |
| 79 | or/24-78 | | | | | 367193 |
| 80 | 4 and 23 and 79 | | | | | 2 |
| 81 | (letter or editorial or comment or news or newspaper article).pt. | | | | | 0 |
| 82 | (addresses or bibliography or biography or case report or comment or congresses or consensus development conference or duplicate publication or editorial or guideline or in vitro or interview or lectures or letter or monograph or news or "newspaper article" or practice guideline or "review literature" or "review of reported cases" or review, academic or review, multicase or review, tutorial or twin study).pt. | | | | | 0 |
| 83 | [animals/ not (humans/ and animals/)] | | | | | 0 |
| 84 | or/81-83 | | | | | 0 |
| 85 | 80 not 84 | | | | | 2 |
| 86 | limit 85 to human [Limit not valid; records were retained] | | | | | 2 |
| 87 | limit 86 to yr="2010 -Current" | | | | | 2 |

*Note: The search strategies provided was designed to assess HRQoL in addition to the work impact of PsA. This manuscript encompasses only those results captured by key terms and synonyms related to work outcomes, as described in Search strategy and selection criteria.*

*Cochrane Database of Systematic Reviews 2005 to October 20, 2021, Database Field Guide EBM Reviews - ACP Journal Club 1991 to September 2021, Database Field Guide EBM Reviews - Database of Abstracts of Reviews of Effects 1^st^ Quarter 2016, Database Field Guide EBM Reviews - Cochrane Clinical Answers September 2021, Database Field Guide EBM Reviews - Cochrane Central Register of Controlled Trials September 2021, Database Field Guide EBM Reviews - Cochrane Methodology Register 3^rd^ Quarter 2012, Database Field Guide EBM Reviews - Health Technology Assessment 4^th^ Quarter 2016, Database Field Guide EBM Reviews - NHS Economic Evaluation Database 1^st^ Quarter 2016; **Search executed on 22 October, 2021 via the Ovid platform.

Table S4. Risk of bias assessment

|  |  | | **Randomized controlled trials**  **NICE Checklist^a^** | | | | | | | | | | | | | | |
| --- | --- | --- | --- | --- | --- | --- | --- | --- | --- | --- | --- | --- | --- | --- | --- | --- | --- |
| Study name, year and NCT | | | | **Selection bias** | |  | | **Performance bias** | | | **Attrition bias** | | |  | | **Detection bias** | |
| KEEPsAKE 1 2022 (NCT03675308)^40^ | | | | Low risk of bias | |  | | Low risk of bias | | | Low risk of bias | | |  | | Low risk of bias | |
| KEEPsAKE 2 2022 (NCT03671148)^41^ | | | | Low risk of bias | |  | | Low risk of bias | | | Low risk of bias | | |  | | Low risk of bias | |
| BE OPTIMAL 2022 (NCT03895203)^39^ (data on file) | | | | Low risk of bias | |  | | Low risk of bias | | | Low risk of bias | | |  | | Low risk of bias | |
| SELECT-PsA 1 2021 (NCT03104400)^22^ | | | | Low risk of bias | |  | | Low risk of bias | | | Unclear | | |  | | Low risk of bias | |
| DISCOVER-2 2021 (NCT03158285)^42^ | | | | Low risk of bias | |  | | Unclear | | | Low risk of bias | | |  | | Unclear | |
| SELECT-PsA 2 2021 (NCT03104374)^43^ | | | | Low risk of bias | |  | | Low risk of bias | | | Unclear | | |  | | Unclear | |
| FUTURE 1 2017 (NCT01392326)^44^ | | | | Low risk of bias | |  | | Low risk of bias | | | Low risk of bias | | |  | | Low risk of bias | |
| SPIRIT-P2 2017 (NCT02349295)^45,46^ | | | | Low risk of bias | |  | | Low risk of bias | | | Low risk of bias | | |  | | Low risk of bias | |
| SPIRIT-P1 2018 (NCT01695239)^45,47^ | | | | Low risk of bias | |  | | Low risk of bias | | | Low risk of bias | | |  | | Low risk of bias | |
| FUTURE 2 2015 (NCT01752634)^48^ | | | | Low risk of bias | |  | | Low risk of bias | | | Low risk of bias | | |  | | Low risk of bias | |
|  | | **Non-randomized studies**  **Newcastle-Ottawa Scale Cohort Studies^b^** | | | | | | | | | | | | | | | |
| **Study name, year and NCT** | | | | **Selection** | | | | | | **Comparability** | | **Outcome** | | | | | **Total**  **(9*)** |
|  |  |  |  | **Representativeness of exposed cohort (*)** | **Selection of non-exposed cohort (*)** | | **Ascertainment of exposure (*)** | | **Outcome not present at start of study (*)** | **(**)** | | **Assessment of outcome (*)** | **Length of follow up (*)** | | **Adequacy of follow up (*)** | |  |
| Nakagawa 2019 (NCT02414633)^38^ | | | | * | – | | – | | * | – | | – | * | | * | | * * * * (4) |
| Corrona 2020 (NCT02530268)^21^ | | | | * | – | | * | | * | – | | – | * | | * | | * * * * * (5) |

*^a^ National Institute for Health and Care Excellence. The guidelines manual: appendices B-I (Appendix C: Methodology checklist: randomised controlled trials).*

*^b^ Wells GA, Shea B, O’Connell D, et al. The Newcastle-Ottawa Scale (NOS) for assessing the quality of nonrandomised studies in meta-analyses.*
